# Single‐Nucleus Transcriptomics Uncovers Xaf1‐Driven PANoptosis as a Therapeutic Target in Aminoglycoside‐Induced Hearing Loss

**DOI:** 10.1111/cpr.70081

**Published:** 2025-07-02

**Authors:** Xinlin Wang, Hairong Xiao, Jiheng Wu, Yanqin Lin, Yiheng Ao, Zixuan Ye, Xin Tan, Fanliang Kong, Xin Chen, Renjie Chai, Shasha Zhang

**Affiliations:** ^1^ Department of Otolaryngology Head and Neck Surgery Zhongda Hospital, State Key Laboratory of Digital Medical Engineering, Jiangsu Provincial Key Laboratory of Critical Care Medicine, School of Life Sciences and Technology, School of Medicine, Advanced Institute for Life and Health, Southeast University Nanjing China; ^2^ Southeast University Shenzhen Research Institute Shenzhen China; ^3^ School of Clinical and Basic Medical Sciences, Shandong Provincial Hospital, Medical Science and Technology Innovation Center Shandong First Medical University & Shandong Academy of Medical Sciences Jinan Shandong China; ^4^ Department of Neurology Aerospace Center Hospital, School of Life Science, Beijing Institute of Technology Beijing China; ^5^ Co‐Innovation Center of Neuroregeneration Nantong University Nantong China; ^6^ Institute for Stem Cell and Regeneration, Chinese Academy of Science Beijing China; ^7^ Beijing Key Laboratory of Neural Regeneration and Repair Capital Medical University Beijing China

**Keywords:** hair cell, neomycin, PANoptosis, snRNA‐seq, Xaf1

## Abstract

Aminoglycoside antibiotics are essential in managing many life‐threatening diseases. However, their derivatives, such as neomycin, are associated with severe side effects such as persistent sensorineural hearing loss. Therefore, it is essential to elucidate the molecular and biochemical mechanisms of aminoglycoside‐induced ototoxicity and identify targets for alleviating ototoxic injury. Here, we provide a detailed cochlear cell atlas of neomycin‐induced acute and chronic ototoxicity‐related changes through single‐nucleus RNA sequencing profiling. Utilising this cochlear cell atlas, we used the *Augur* and *scDist* algorithms to evaluate cell‐type‐specific susceptibility to neomycin injury. We observed aberrant expression of X‐linked inhibitor of apoptosis (Xiap)–associated factor 1 (Xaf1) in neomycin‐exposed cochleae using the cochlear cell atlas, and we identified a novel role for Xaf1 in facilitating PANoptosis through overexpression and knockdown assays in vitro. Finally, we assessed the protective role of Xaf1 against neomycin‐induced ototoxicity by *Xaf1* knockdown in cochlear hair cells using adeno‐associated virus‐based gene delivery. Mechanistically, Xaf1 orchestrates PANoptosis activation through direct interaction with and transcriptional regulation of ZBP1, establishing its hierarchical position upstream in the signalling cascade. This study presents detailed cochlear cellular maps of neomycin‐induced ototoxicity and serves as a valuable resource for identifying transcriptome‐wide disease‐driving perturbations at the single‐cell level. More importantly, we identified Xaf1 as a critical target for modulating the PANoptosis pathway, offering a promising treatment strategy for aminoglycoside‐induced ototoxicity.

## Graphical abstract

Neomycin induces increased expression of Xaf1 in cochlear HCs, triggering PANoptosis, which encompasses pyroptosis, apoptosis and necroptosis (left). Targeted *Xaf1* knockdown in HCs through gene therapy can significantly inhibit the occurrence of PANoptosis in HCs (right).

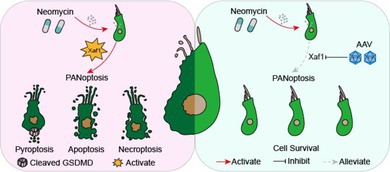

## 1 Introduction

Approximately 20% of the global population suffers from some degree of hearing loss (HL), with 430 million individuals experiencing substantial hearing impairment (classified as HL over 40 dB), as reported by the World Health Organisation [1]. Sensorineural HL (SNHL) arises from both structural and functional damage to the cochlea, predominantly impacting spiral ganglion neurons (SGNs), hair cells (HCs) and the stria vascularis (SV) [2]. Unlike other forms of HL, SNHL is generally irreversible, accounting for approximately 90% of all documented cases of hearing impairment [3]. Well‐established risk factors for SNHL include aging, exposure to loud noise and the use of ototoxic medications [2, 4].

Aminoglycosides (AGs), as well as many non‐aminoglycoside antibiotics, salicylates and anticancer medicines, constitute the most commonly encountered ototoxic pharmaceuticals. AGs are water‐soluble, broad‐spectrum antibiotics primarily used to treat Gram‐negative bacterial infections [5]. They are frequently used in neonatal critical care facilities, where up to 80% of infants receive empirical or preventative therapies [6, 7]. AGs are also used in the treatment of several life‐threatening illnesses, including urinary tract infections, tuberculosis‐related infections, sepsis and immunodeficiency [8]. However, AGs, particularly neomycin, are associated with significant risks, such as reversible nephrotoxicity, irreversible neurotoxicity and SNHL [8, 9]. Despite these adverse effects, AGs remain a cornerstone of therapy due to their efficacy against illnesses caused by multidrug‐resistant bacteria [10, 11]. While the antimicrobial potency of AGs remains clinically irreplaceable, their deployment continues to be plagued by intractable ototoxic sequelae that demand urgent therapeutic responses.

Recent advances in the understanding of AG ototoxicity have revealed key mechanisms underlying this form of cochlear damage, including the production of reactive oxygen species (ROS), excessive activation of glutamatergic receptors (notably N‐methyl‐D‐aspartate receptors) and the impairment of mitochondrial protein synthesis [12]. In the inner ear, AGs induce free radical formation, which causes irreversible damage to HCs and SGNs, ultimately leading to HL [13], and histopathological studies indicate that outer hair cells (OHCs) exhibit greater susceptibility to ototoxic damage compared to inner hair cells (IHCs) [14]. Interestingly, the histological damage observed in animal models is more consistent with apoptosis than necrosis [15], and apoptosis is predominantly governed by caspase activation, which can be initiated via intrinsic and extrinsic mechanisms [16]. Clarifying the cellular signalling pathways that cause AG‐induced HC loss is of growing interest [13]. However, it remains unclear whether AG‐induced HC death involves mechanisms beyond apoptosis. Additionally, the relative susceptibility of different cochlear cell types to AG‐induced damage is not yet fully understood. To prevent the ototoxicity of AGs, it is crucial to have a comprehensive understanding of the molecular and biochemical mechanisms of neomycin‐induced cochlear damage and to identify critical molecular targets that can alleviate cochlear injury.

In this study, we sought to comprehensively clarify the molecular and biochemical pathways underlying AG‐induced cochlear injury and to identify key targets involved in regulating cochlear injury. Thus, we conducted single‐nucleus RNA sequencing (snRNA‐seq) on neomycin‐induced acute and chronic cochlear ototoxicity models. Using single‐cell transcriptomics, we generated two detailed cochlear injury single‐cell atlases—one for acute neomycin‐induced injury, encompassing cochlear epithelium cell types, and another for chronic injury, comprising total cochlear cell types. Comparative analysis of these atlases revealed that HCs, SGNs and SV cells exhibit heightened susceptibility to neomycin‐induced damage. Notably, we observed a distinct form of PANoptosis in neomycin‐damaged HCs, wherein apoptosis occurs concurrently with necroptosis and pyroptosis. Furthermore, we identified a previously uncharacterised role for the X‐linked inhibitor of apoptosis (Xiap)‐associated factor 1 (Xaf1) in promoting PANoptosis in HCs during neomycin‐induced cochlear injury. We further demonstrated that *Xaf1* knockdown considerably decreased neomycin‐induced HL in mice, highlighting its potential as a therapeutic target for AG‐induced ototoxicity. Combining co‐immunoprecipitation and functional interrogation, we conclusively demonstrated that Xaf1 directly engages Zbp1 through physical interaction, mechanistically coupling this complex to PANoptosis activation.

## 2 Results

### 2.1 Cell Atlas of AG‐Dependent Acute Cochlear Epithelial Injury

**FIGURE 1:**
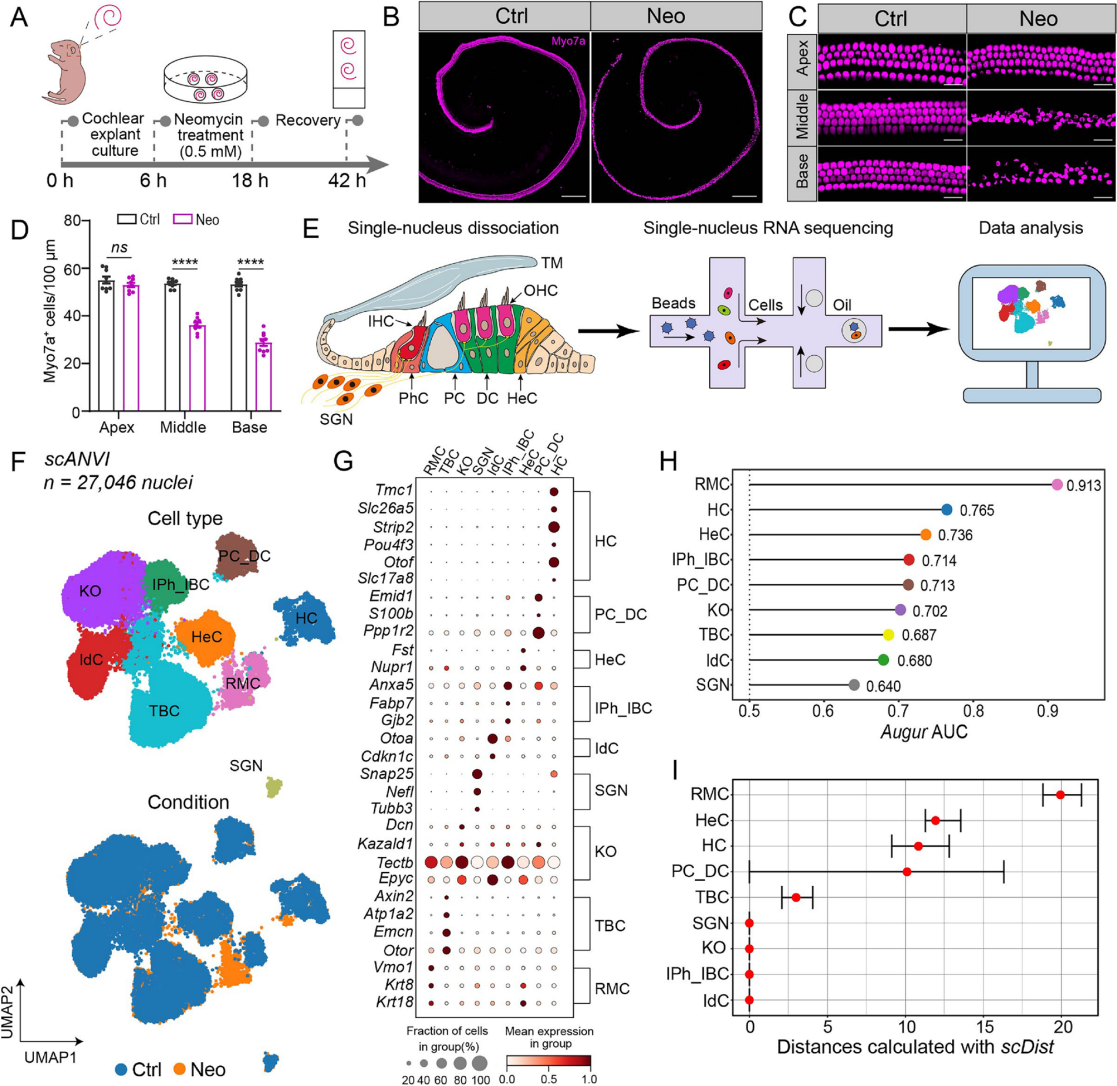
Single‐nucleus transcriptomic profiling reveals cochlear cell‐type‐specific vulnerability to neomycin‐induced acute ototoxicity injury. (A) The schematic workflow for creating the neomycin‐induced acute ototoxicity model. (B) Cochlear HC survival was analysed by anti‐Myo7a immunolabelling in the Ctrl and Neo groups. Scale bar, 200 μm. (C‐D) Regional quantification (C) and quantitative analysis (D) of Myo7a^+^ HCs in the apical, middle and basal cochlear turns. Scale bar, 20 μm in (C). *n* = 9 in (D). (E) The experimental paradigm for snRNA‐seq in the acute ototoxicity model. (F) Integrative UMAP visualisation of 27,046 nuclei across the Ctrl and Neo groups in the acute ototoxicity model. Top: Canonical marker‐defined cochlear cell clusters (HC: Hair cells; IPh/IBC: Inner phalangeal/border cells; PC/DC: Pillar/Deiters' cells; HeC: Hensen's cells; IdC: Interdental cells; KO: Kölliker's organ; TBC: Tympanic border cells; SGN: Spiral ganglion neurons; RM: Reissner's membrane). Bottom: Cell embeddings of the Ctrl and Neo group maps. (G) The dot plot shows cell‐type‐specific expression patterns in the acute ototoxicity model. (H) *Augur* algorithm‐derived AUC scores predicted the vulnerability to neomycin‐induced acute ototoxicity injury across nine cochlear cell types. (I) The *scDist* trajectory analysis showed differential vulnerability across cell types in the neomycin‐induced acute ototoxicity model. ns, no significance. ****p < 0.0001.

To fully delineate the pathogenic cascades activated upon neomycin‐induced ototoxicity, we performed snRNA‐seq of cochlear explants exposed to neomycin. Following established ototoxicity induction protocols, cochlear explants were incubated with 0.5 mM neomycin for 12 h in defined culture conditions, with a subsequent 24 h drug‐free recovery to capture acute phase pathophysiological responses (Figure 1A). Quantitative analysis of Myosin VIIa (Myo7a)‐stained cochlear whole‐mount preparations revealed a marked reduction in Myo7a‐positive HCs (Myo7a^+^ HCs) in the neomycin‐treated (Neo) group compared to the control (Ctrl) group. Consistent with established ototoxicity patterns, it was predominantly HCs in the middle and basal cochlear turns that were lost, reinforcing the well‐documented increased vulnerability of the cochlear basal regions to aminoglycoside toxicity (Figure 1B–D). Next, we used the 10× Genomics platform to systematically profile the transcriptomes of neomycin‐induced injured cochlear explants to map the effects on different cell types upon neomycin exposure (Figure 1E). Using rigorous quality controls (Figure S1A–C) and canonical marker‐based annotation [17], we profiled 27,046 high‐quality nuclei spanning nine distinct cell types (Ctrl group: 16,366 nuclei, Neo group: 10,680 nuclei) (Figure 1F,G). To effectively account for technical batch effects while preserving the biological signals, we systematically benchmarked six integration algorithms (*Scanorama*, *BBKNN*, *Harmony*, *ComBat*, *scVI* and *scANVI*) (Figure S1D and Figure 1F). The comparative evaluation showed that *scANVI* was superior at preserving biologically meaningful variations while effectively mitigating technical artefacts (Figure S1E). Leveraging this optimised framework, we implemented the *Augur* and *scDist* algorithms to quantify cell‐type‐specific vulnerability to neomycin injury (Figure 1H,I). Both algorithms identified Reissner's membrane cells, HCs and Hensen's cells as the most sensitive populations in the acute neomycin‐injury model, and these cell types exhibited pronounced transcriptional alterations upon neomycin exposure.

### 2.2 Cell Atlas of AG‐Dependent Chronic Cochlear Injury

The mammalian cochlea comprises four functionally distinct compartments: (1) the sensory epithelium that contains mechanosensory IHCs and OHCs with associated supporting cells (SCs), (2) SGNs associated with glial cells (GCs), (3) the SV and (4) fibroblasts that are firmly attached to the SV [18]. IHCs transmit auditory signals via ribbon synapses to type I SGNs, while OHCs enhance sound sensitivity through electromotility [19]. SCs exhibit glia‐like functions, including ionic homeostasis and structural maintenance [20]. The SV maintains the endocochlear potential through potassium ions secreted by three layers of cellular structures (marginal, intermediate and basal cells) [21]. In the SV, mutated genes related to ion channels and their regulatory subunits (e.g., *KCNE1*, *KCNQ1* and *KCNJ10*) may lead to hearing impairment [22]. Therefore, it is necessary to evaluate the response of SV cells to AG stimulation and improve our current cellular atlas of AG‐induced acute cochlear injury.

**FIGURE 2:**
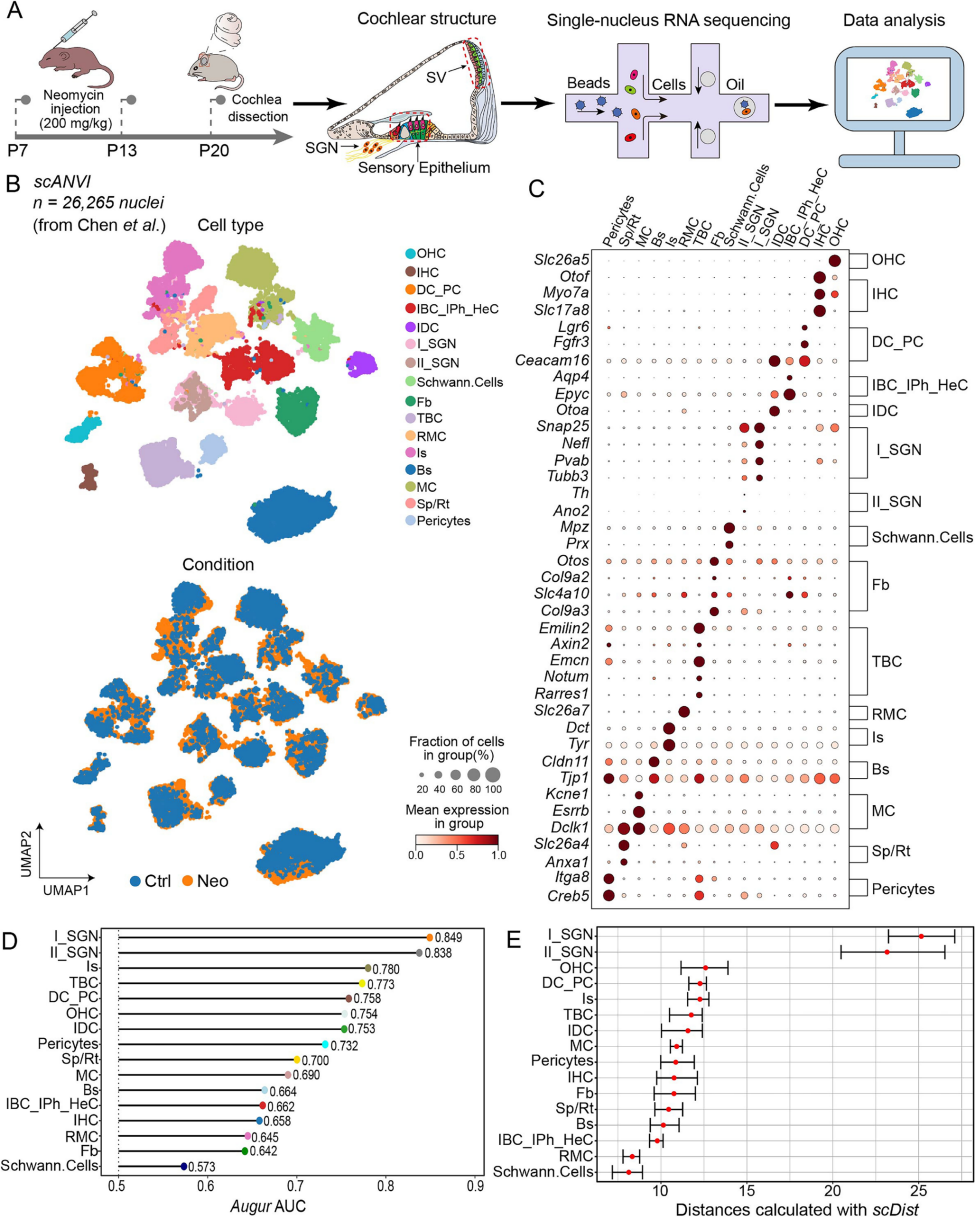
Single‐nucleus transcriptomic profiling reveals cochlear cell‐type‐specific vulnerability to neomycin‐induced chronic ototoxicity injury. (A) Schematic workflow for snRNA‐seq of the neomycin‐induced chronic ototoxicity model. (B) Integrative UMAP visualisation of 26,265 nuclei across the Ctrl and Neo groups in the chronic ototoxicity model. Top: Cell‐type classification (IBC_IPh_HeC: Inner border/phalangeal/Hensen's cells; Fb: Fibrocytes; Is: Intermediate stria cells; Bs: Basal stria cells; MC: Marginal stria cells; Sp/Rt: Spindle/Root cells). Bottom: Cell embeddings of the Ctrl and Neo groups map. (C) The dot plot shows cell‐type‐specific expression patterns in the chronic ototoxicity model. (D) *Augur* algorithm‐derived AUC scores predicted vulnerability across 16 cochlear cell types to neomycin‐induced ototoxicity. (E) The *scDist* trajectory analysis showed differential vulnerability across cochlear cell types in the neomycin‐induced chronic ototoxicity model.

To systematically evaluate the cell‐type‐specific susceptibility of cochlear cells to neomycin, we reanalysed previously published snRNA‐seq data of neomycin‐induced chronically injured cochleae [23]. Following rigorous quality control (Figure S2A–B) and canonical marker‐based annotation [24], we profiled 26,265 high‐quality nuclei spanning 16 distinct cell types (Ctrl group: 6598 nuclei, Neo group: 19,667 nuclei) (Figure 2B,C). Next, we implemented six batch‐correction algorithms (*Scanorama*, *BBKNN*, *Harmony*, *ComBat*, *scVI*, *scANVI*) to optimise biological signal preservation (Figure S2C). The comparative evaluation identified *scANVI* as the superior integration method, as quantified by cluster resolution and batch‐mixing metrics (Figure S2D). The susceptibility of various cell types to neomycin injury was evaluated using *Augur* and *scDist*, and these identified the vulnerability of OHCs, type I/II SGNs and intermediate cells in the cochlear epithelium, modiolus and SV regions (Figure 2D,E). To delineate the biological consequences of neomycin exposure on cochlear hair cells, we conducted GO enrichment analysis of DEGs comparing the Ctrl and Neo groups. Comparative analysis of acute neomycin‐induced damage revealed significant enrichment of biological processes associated with sensory organ development and stereocilium bundle in the Neo group (Figure S3A–B). In contrast, chronic neomycin exposure preferentially enriched pathways governing negative regulation of epithelial cell proliferation and regulation of cell growth (Figure S3C–D).

### 2.3 PANoptosis Occurred in the Neomycin‐Exposed Mouse Cochlea

**FIGURE 3:**
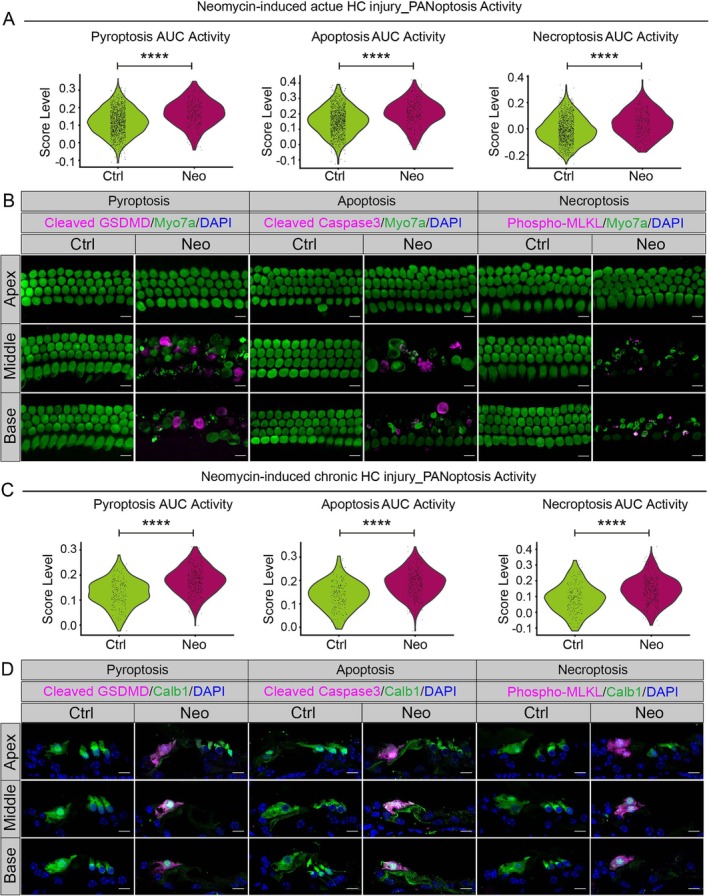
HCs undergo PANoptosis in neomycin‐induced ototoxicity. (A) AUC scores for PANoptosis‐associated programmed cell death pathways (pyroptosis, apoptosis, necroptosis) in the Ctrl and Neo groups from the snRNA‐seq analysis of the neomycin‐induced acute ototoxicity model. (B) Cochlear explant immunofluorescence revealed co‐localisation of Myo7a^+^ HCs with PANoptosis markers (Cleaved GSDMD, pyroptosis marker; Cleaved Caspase3, apoptosis marker; Phospho‐MLKL, necroptosis marker). Scale bars, 10 μm. (C) Same as in (A), but for the neomycin‐induced chronic ototoxicity model. (D) Immunofluorescence of cochlear sections revealed co‐localisation of Myo7a^+^ HCs and PANoptosis markers in the neomycin‐induced chronic ototoxicity model. Scale bars, 10 μm. ****p < 0.0001.

Recent studies on the ototoxic effects of AGs have identified essential biochemical and physiological pathways, including ROS production, abnormal calcium influx, lipid peroxidation, disruption of mitochondrial protein synthesis and hyperactivation of glutamatergic receptors [5, 25]. Disruption of these metabolic processes can result in the death of HCs and SGNs, and histopathological analyses have suggested that AGs predominantly induce apoptosis in HCs and SGNs rather than necrosis [15]. It is currently unclear whether AG‐induced cochlear cell mortality entails mechanisms other than apoptosis. Utilising snRNA‐seq, we demonstrated that neomycin triggers PANoptosis in HCs both in vitro and in vivo (Figure 3A,C), which is in contrast to the previously reported apoptosis induction by AGs. PANoptosis is a recently identified inflammatory programmed cell death pathway that is regulated by the assembly of PANoptosome complexes, and it involves the intricate interplay and coordination among pyroptosis (P), apoptosis (A) and necroptosis (N) [26]. Apoptosis is molecularly regulated by the activation of executioner caspases, including caspase3 and caspase7, which are downstream of the initiator caspases, including caspase8, caspase9 and caspase10 [27, 28, 29]. Pyroptosis is initiated by the creation of plasma membrane pores mediated by Gasdermin‐family proteins, with Gasdermin D (GSDMD) serving as the archetypal effector, and these proteins are activated by inflammatory Caspase1 and Caspase11 in mice or by CASPASE4/5 in humans [30, 31]. The execution of necroptosis is mediated through MLKL pseudo‐kinase domain oligomerisation into membrane‐perforating supramolecular complexes after RIPK3‐mediated phosphorylation within the RIPK1‐RIPK3 signalosome activation platform [32, 33, 34]. In addition, immunofluorescence staining of Cleaved GSDMD (a pyroptosis marker), Cleaved Caspase3 (an apoptosis marker) and Phospho‐MLKL (a necroptosis marker) confirmed that neomycin‐induced acute and chronic ototoxicity resulted in PANoptosis in HCs (Figure 3B,D). Meanwhile, at single‐cell resolution, neomycin was also shown to induce PANoptosis in SV cells in vivo (Figure S4A–B). However, neomycin only induced pyroptosis and apoptosis in injured type I SGNs, and no necroptosis was observed in vivo (Figure S4C–D). The above results indicated that HCs, type I SGNs and SV cells have different responses to neomycin injury, and the PANoptosis induced by neomycin‐induced ototoxicity exhibits cellular heterogeneity.

### 2.4 Xaf1 Expression Was Increased in HCs in the Neomycin‐Induced Ototoxicity Model

The primary mechanism responsible for AG‐induced ototoxicity is the activation of caspase‐mediated apoptotic pathways in the cochlea [5]. Xiap exerts an anti‐apoptotic effect by directly binding to activated caspase [35], and Xaf1 was initially identified as a natural antagonist of Xiap, counteracting its anti‐caspase function by inhibiting the RING domain of Xiap [35]. Prior scRNA‐seq findings revealed significant upregulation of *ZBP1*, *XAF1*, *IFI44L* and *SOCS1* in B cells from sepsis patients that exhibit increased susceptibility to PANoptosis [36]. Given these studies, we speculated that the critical biochemical mechanism of neomycin‐induced ototoxicity includes the promotion of PANoptosis in HCs by Xaf1.

**FIGURE 4:**
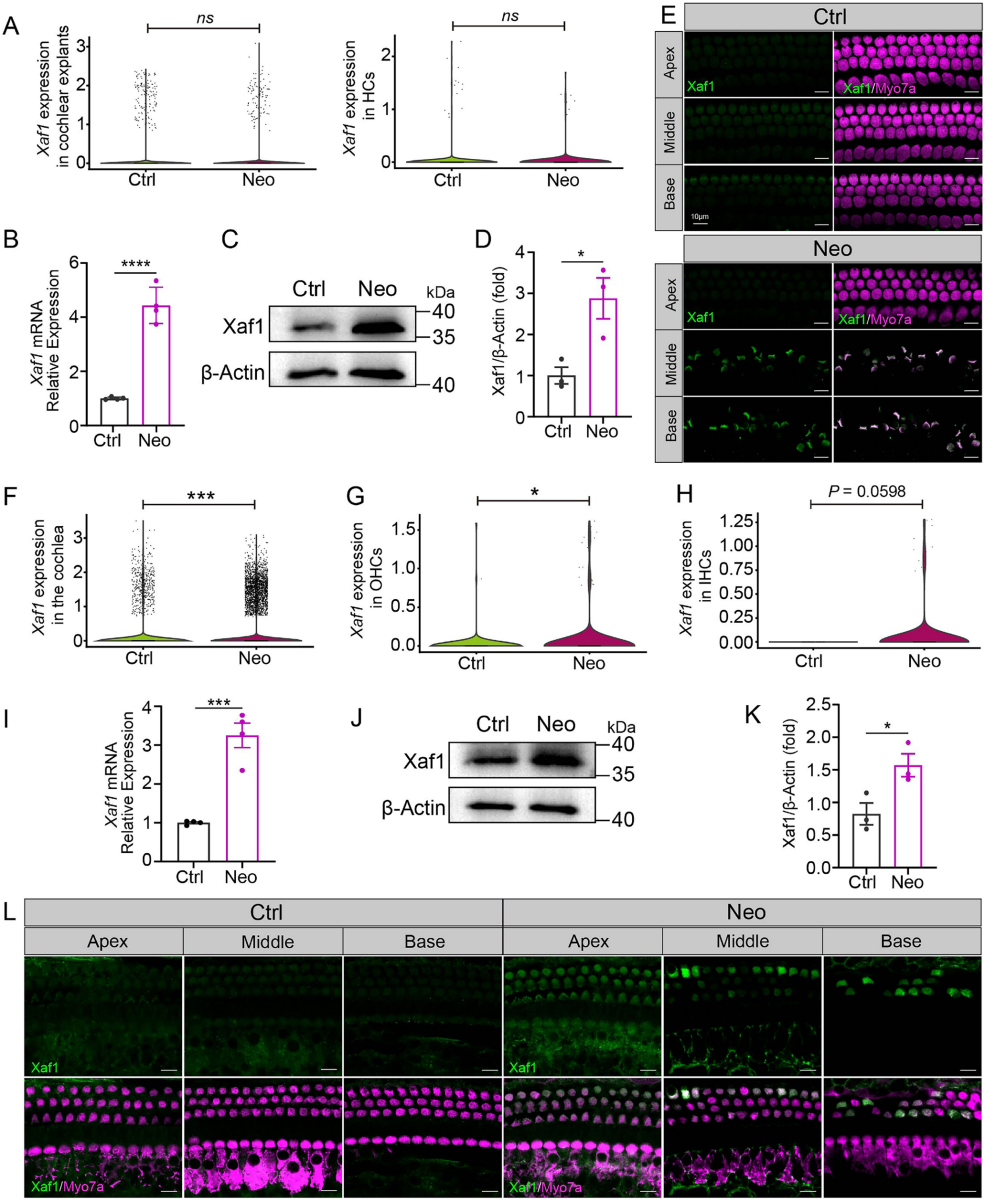
Xaf1 expression is upregulated in neomycin‐induced cochlear HCs. (A) SnRNA‐seq analysis of neomycin‐treated cochlear explants revealed *Xaf1* expression in cochlear explants and HCs. (B) RT‐qPCR showed an increase in the *Xaf1* mRNA level in the Neo group. (C‐D) The western blot showed increased Xaf1 protein levels in the Neo group (C) and the protein quantification is shown in (D), *n* = 3. (E) Whole‐mount cochlear immunofluorescence showed Xaf1‐Myo7a co‐localisation in the neomycin‐induced acute ototoxicity model. Scale bars, 20 μm. (F‐H) Chronic neomycin administration induced *Xaf1* upregulation in the cochlea (F), OHCs (G) and IHCs (H). (I) RT‐qPCR showed increased *Xaf1* mRNA levels in the Neo group. (J‐K) Western blot showed elevated Xaf1 protein levels in the Neo group (J), and the quantification is shown in (K), *n* = 3. (L) Immunofluorescence of cochlear sections showed Xaf1 and Myo7a co‐localisation in the neomycin‐induced chronically injured cochlea (125 mg/kg × 8 days). Scale bars, 10 μm. ns, no significance. *p < 0.05, ***p < 0.001, ****p < 0.0001.

We explored the involvement of Xaf1 in neomycin‐induced ototoxicity by measuring *Xaf1* expression in acute and chronic cochlear damage models using a snRNA‐seq atlas. In the acutely damaged cochlear explants, the snRNA‐seq analysis showed no significant changes in *Xaf1* expression level within the cochlear explants or in HCs of the Neo group (Figure 4A), but there was a slightly increasing trend in the Neo group. Real‐time quantitative PCR (RT‐qPCR) showed a notable increase in *Xaf1* mRNA levels across the cochlear explants in the Neo group (Figure 4B). In addition, the Xaf1 protein level in the Neo group was increased by at least 1.5‐fold compared to the Ctrl group (Figure 4C,D). Immunofluorescence staining further demonstrated that Xaf1 expression was increased in damaged HCs of the Neo group, and Xaf1 expression co‐localised with Myo7a (Figure 4E).

The snRNA‐seq data analysis indicated a significant increase in *Xaf1* expression levels throughout the chronically injured cochlea and in OHCs in the Neo group compared to the Ctrl group (Figure 4F–H). The RT‐qPCR results confirmed the upregulation of *Xaf1* mRNA levels across the cochlea in the Neo group (Figure 4I). Western blot analysis corroborated these findings, indicating that the Xaf1 protein level in the Neo group was at least one‐fold greater compared to the Ctrl group (Figure 4J,K). Immunofluorescence staining revealed that in the Neo group, Xaf1 expression was increased in the apical, middle and basal cochlear turns (Figure 4L). Together, these results showed that Xaf1 is a key executioner of HC death, thus linking it to the pathology of AG ototoxicity.

### 2.5 Xaf1 Promoted the Occurrence of PANoptosis In Vitro

**FIGURE 5:**
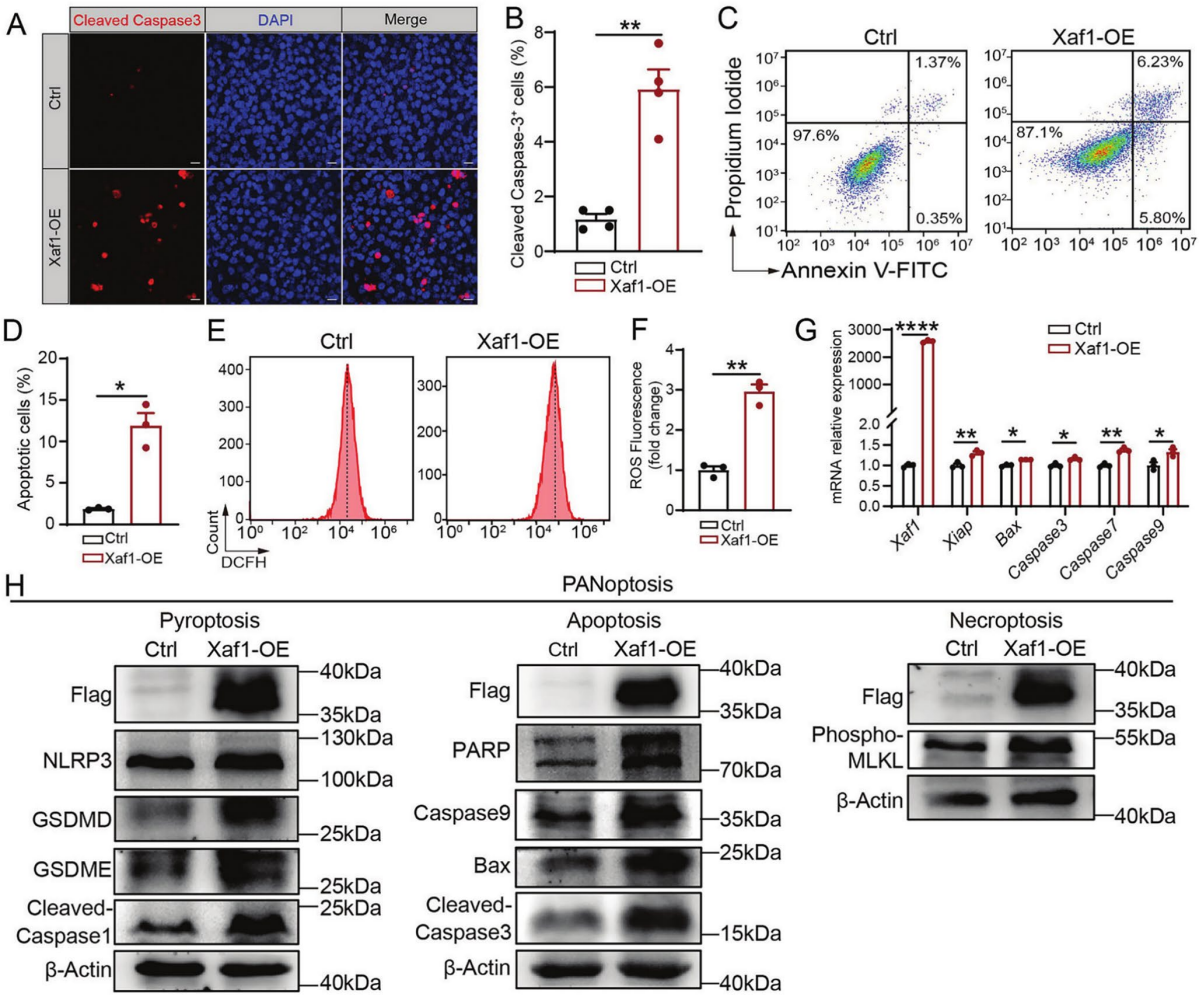
Xaf1 Overexpression triggers PANoptosis in HEI‐OC1 cells. (A) Representative immunofluorescence images of Cleaved Caspase3 (red) and DAPI nuclear counterstain (blue) in the Ctrl and Xaf1‐OE groups. Scale bar, 20 μm. (B) Quantitative analysis of Cleaved Caspase3‐positive cells, *n* = 4. (C) Flow cytometric analysis of apoptosis using Annexin V/PI staining. Quadrant analysis distinguished viable cells (Q3: Annexin V^−^/PI^−^), early apoptotic cells (Q4: Annexin V^+^/PI^−^) and late apoptotic cells (Q2: Annexin V^+^/PI^+^). (D) Statistical quantification of total apoptotic cells (Q2 + Q4), *n* = 3. (E) Flow cytometry profiles showing intracellular ROS level detected by the DCFH‐DA probe. (F) Mean fluorescence intensity quantification of ROS production, *n* = 3. (G) RT‐qPCR analysis of apoptosis‐associated gene expression (*Xaf1*, *Xiap*, *Bax*, *Caspase3*, *Caspase7* and *Caspase9*) following Xaf1 overexpression. (H) Western blot of PANoptosis pathway‐related proteins. β‐Actin served as the loading control. *n* = 3. *p < 0.05, **p < 0.01, ****p < 0.0001.

Prior studies have shown the pro‐apoptotic role of Xaf1 [37, 38], but its role in triggering PANoptosis remains unclear. To address this, we investigated the role of Xaf1 in the initiation of PANoptosis by overexpressing Xaf1 in HEI‐OC1 cells (a mouse cochlear HC line). Immunofluorescence showed a substantial increase in Cleaved Caspase3‐positive cells in the Xaf1 overexpression (Xaf1‐OE) group compared to the Ctrl group (Figure 5A,B). Flow cytometry analysis of apoptosis using Annexin V/PI staining revealed a significantly increased proportion of apoptotic cells in the Xaf1‐OE group (Figure 5C,D), and this was accompanied by a dramatic elevation in ROS levels (Figure 5E,F). Moreover, Xaf1 overexpression markedly enhanced the levels of critical apoptotic genes, specifically *Caspase3*, *Caspase7*, *Caspase9* and *Bax*. (Figure 5G), which was consistent with its pro‐apoptotic function. To further explore the involvement of Xaf1 in PANoptosis, we assessed the classical markers of the PANoptosis signalling pathway, including the pyroptosis pathway (GSDMD, GSDME, NLRP3 and Cleaved Caspase1), the apoptosis pathway (Cleaved Caspase3, Cleaved Caspase9, PARP and Bax) and the necroptosis pathway (Phospho‐MLKL), using Western blot analysis. Importantly, the Xaf1‐OE group exhibited significant upregulation of these PANoptosis markers compared to the Ctrl group (Figure 5H). Our findings collectively suggest a novel role for Xaf1 in promoting both pyroptosis and necroptosis, underscoring its distinct function in initiating PANoptosis.

### 2.6 Xaf1 Is Essential for PANoptosis of HCs During Neomycin‐Induced Ototoxicity

**FIGURE 6:**
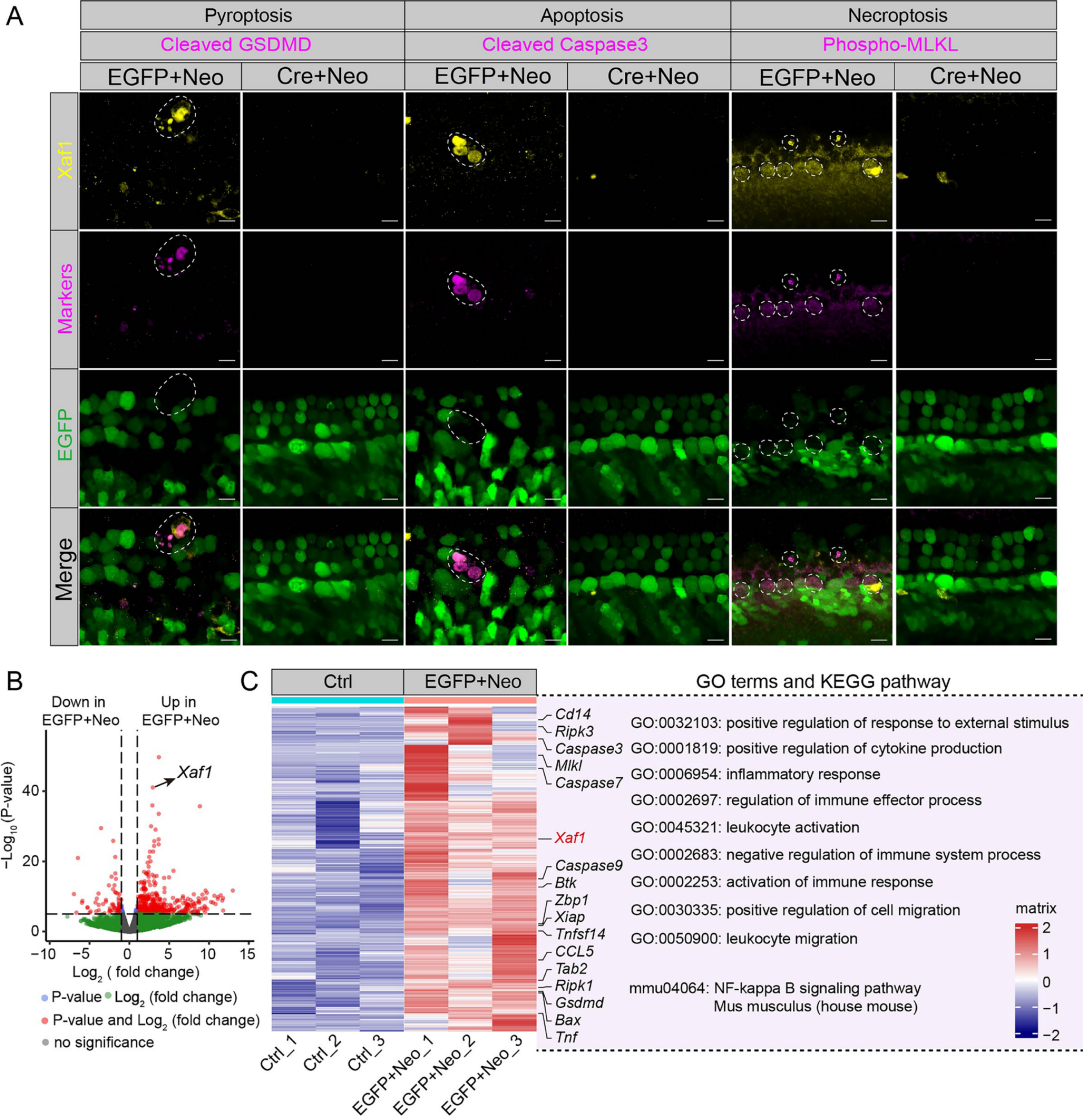
Xaf1 is crucial for neomycin‐induced PANoptosis of HCs. (A) Immunofluorescence staining for Xaf1 (yellow), Cleaved GSDMD (a pyroptosis marker, magenta), Cleaved Caspase3 (an apoptosis marker, magenta), Phospho‐MLKL (a necroptosis marker, magenta) and EGFP (green) in the middle turn of cochlear explants in the EGFP+Neo and Cre + Neo groups. Scale bar, 10 μm. (B) The volcano plot shows the differentially expressed genes (DEGs) between the Ctrl and EGFP+Neo groups. (C) GO analysis of the upregulated genes in the EGFP+Neo group using the *Metascape* platform.

To investigate the role of Xaf1 in PANoptosis activation during neomycin‐induced ototoxicity, we examined the co‐localisation of Xaf1 and PANoptosis markers in the neomycin‐induced injured cochlear explants. We injected adeno‐associated viruses (AAVs), including AAV‐EGFP and AAV‐Cre, into postnatal day (P)1 *Xaf1* ^flox/flox^ mice, followed by continuous injection of neomycin at P7 for 8 days (referred to as the EGFP+Neo group and Cre + Neo group hereafter, respectively). Immunofluorescence results confirmed that Xaf1‐positive cells co‐localised with Cleaved GSDMD, Cleaved Caspase3 and Phospho‐MLKL predominantly within the EGFP‐marked HC region in the EGFP+Neo group. In contrast, the Cre + Neo group showed decreased Xaf1 expression and the absence of PANoptosis markers in cochlear explants (Figure 6A). These findings demonstrated that *Xaf1* knockdown in HCs suppresses PANoptosis, highlighting its essential role in PANoptosis activation following neomycin‐induced ototoxicity.

To explore the mechanism underlying Xaf1‐mediated PANoptosis inhibition, we performed bulk RNA sequencing (RNA‐seq) on cochlear tissues from the Ctrl and EGFP+Neo groups. Principal component analysis (PCA) of RNA‐seq data demonstrated pronounced transcriptional segregation between Ctrl and EGFP+Neo groups along the primary variance axis (Figure S6A), indicative of neomycin‐induced global transcriptomic remodelling. The EGFP+Neo group showed downregulation of 2006 genes and upregulation of 2863 genes compared to the Ctrl group (Figure 6B). Notably, the level of *Xaf1* was markedly increased in the EGFP+Neo group compared to the Ctrl group (Figure 6B). Subsequently, we performed Gene Ontology (GO) and Kyoto Encyclopedia of Genes and Genomes (KEGG) pathway analysis on these upregulated genes using the *Metascape* platform to explore the mechanism of neomycin‐induced ototoxicity. The upregulated genes in the EGFP+Neo group were mainly involved in inflammatory responses and immune system activation (Figure 6C). Prior research showed that stimulation of the NF‐κB signalling pathway exerts a significant proinflammatory effect [39]. The activation‐related genes of the NF‐κB signalling pathway, such as *Btk*, *Cd14*, *Tnf*, *Tnfsf14*, *Tab2* and *CCL5*, were significantly increased in the EGFP+Neo group (Figure 6C). More importantly, PANoptosis‐related genes, including the pyroptosis pathway (*Gsdmd*), apoptosis pathway (*Caspase3*, *Caspase7*, *Caspase9* and *Bax*) and necroptosis pathway (*Ripk1*, *Ripk3* and *Mlkl*), were significantly enriched in the EGFP+Neo group (Figure 6C). Notably, *Xaf1* and *Zbp1*, which are crucial genes governing the manifestation of PANoptosis, were markedly increased in the EGFP+Neo group (Figure 6C). *Zbp1* promotes the occurrence of PANoptosis by participating in the formation of the PANoptosome complex [40], which is consistent with our results. In summary, neomycin triggers PANoptosis of HCs through Xaf1 upregulation, and we found that targeted *Xaf1* knockdown in HCs via gene therapy could attenuate PANoptosis.

### 2.7 *Xaf1* Knockdown Alleviated Neomycin‐Induced HC Injury and Maintained Hearing Function

**FIGURE 7:**
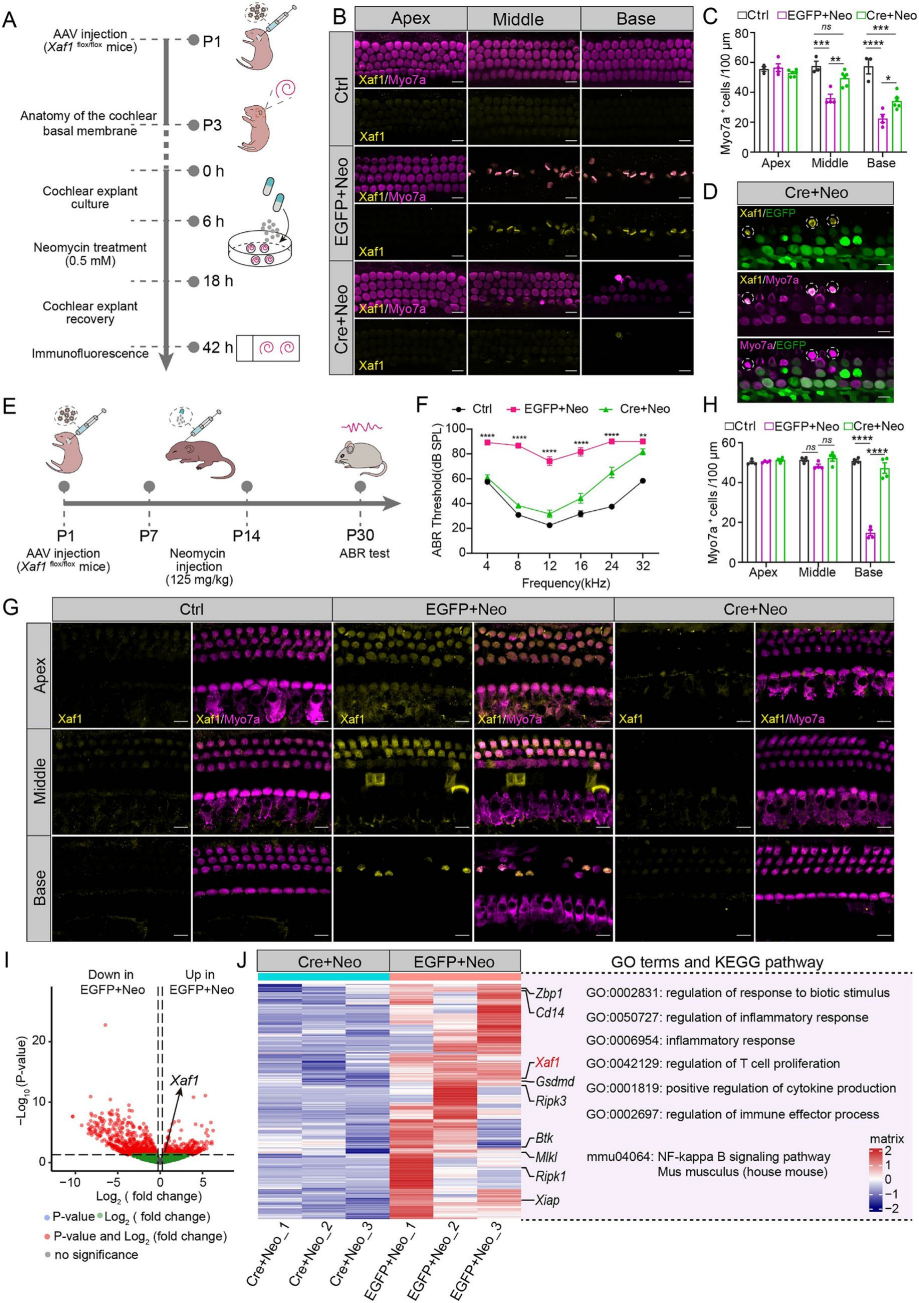
*Xaf1* knockdown attenuates neomycin‐induced HC injury in vitro and in vivo. (A) Experimental design for neomycin‐induced cochlear explant injury in vitro. Cochleae from *Xaf1* ^flox/flox^ mice (P1) were injected with AAV‐EGFP or AAV‐Cre. After a 48‐h recovery, the explants were treated for 12 h with 0.5 mM Neo, followed by a 24 h recovery. (B) Immunofluorescence staining showing Xaf1 (yellow) and the HC marker Myo7a (magenta) in the cochlear epithelial tissues. Scale bar, 10 μm. (C) Quantification of Myo7a^+^ HCs in cochlear explants (Ctrl group: *n* = 3; EGFP+Neo group: *n* = 4; Cre + Neo group: *n* = 6). (D) AAV‐mediated *Xaf1* knockdown efficiency validation in cochlear explants. (E) Experimental design in vivo. *Xaf1* ^flox/flox^ mice (P1) were injected with AAV‐EGFP or AAV‐Cre, followed by 8 days of subcutaneous neomycin treatment (125 mg/kg/day). (F) ABR tests in the Ctrl, EGFP+Neo and Cre+Neo groups, respectively (*n* = 6). Statistical analysis compared the Cre + Neo group to the EGFP+Neo group. (G) Immunofluorescence staining of XAF1 (yellow) and Myo7a (magenta) in the Ctrl, EGFP+Neo and Cre + Neo groups, respectively. Scale bar, 10 μm. (H) Quantitative analysis of Myo7a^+^ cells in the cochlear turns of the neomycin‐induced ototoxicity model in vivo (*n* = 4). (I) The volcano plot shows the DEGs between the EGFP+Neo and Cre+Neo groups. (J) GO analysis of the upregulated genes in the EGFP+Neo group using the *Metascape* platform. ns, no significance. *p < 0.05, **p < 0.01, ***p < 0.001, ****p < 0.0001.

Next, we asked whether *Xaf1* knockdown could prevent HC loss from neomycin exposure, and we injected AAV‐Cre into *Xaf1* ^flox/flox^ mice to knock down *Xaf1*. In the neomycin‐induced acute injury model in vitro, *Xaf1* ^flox/flox^ mice (P1) were injected with AAV‐EGFP or AAV‐Cre through the round window membrane, and cochlear basilar membranes were dissected at P3 for cochlear explant culture. Cochlear explants underwent treatment with 0.5 mM neomycin for 12 h, followed by a 24 h recovery phase (Figure 7A). The AAV infection efficiency of HCs in cochlear explants of the Cre + Neo group exceeded 80% (Figure S5A–B). Immunofluorescence showed considerably increased Xaf1 expression in the middle and basal turns of the EGFP+Neo group alongside a substantial decrease in Myo7a^+^ HCs. In contrast, the Cre+Neo group showed decreased Xaf1 expression and a significant increase in Myo7a^+^ cells compared to the EGFP+Neo group (Figure 7B,C). Notably, in the Cre+Neo group, the EGFP‐uninfected HCs displayed high Xaf1 levels, whereas EGFP‐infected HCs exhibited decreased Xaf1 levels, suggesting a protective role of *Xaf1* knockdown against neomycin‐induced injury (Figure 7D).

In the neomycin‐induced chronic injury model in vivo, *Xaf1* ^flox/flox^ mice (P1) received injections of AAV‐EGFP or AAV‐Cre, followed by subcutaneous neomycin administration (125 mg/kg) from P7 to P14, and auditory brainstem response (ABR) tests were performed at P30 (Figure 7E). The AAV infection efficiency of HCs in the cochleae from the Cre group exceeded 85% (Figure S5C–D). ABR results showed that the EGFP+Neo group exhibited a hearing threshold increase of ≥ 20 dB across low (4 kHz and 8 kHz), middle (12 kHz and 16 kHz) and high (24 kHz and 32 kHz) frequencies compared to the Ctrl group, indicating that neomycin induced serious HL (Figure 7F). In contrast, the Cre + Neo group showed significantly better hearing across almost all hearing frequencies, largely similar to the Ctrl group from low to middle frequencies (4 kHz–16 kHz), suggesting robust hearing protection (Figure 7F). Immunofluorescence showed that the EGFP+Neo group displayed increased Xaf1 expression in the apical, middle and basal cochlear turns, accompanied by a significant decrease in HCs in the basal turn (Figure 7G,H). Notably, the Cre + Neo group showed decreased Xaf1 expression across all cochlear turns and a marked increase in basal turn HCs (Figure 7G,H). These findings suggested that *Xaf1* knockdown in cochlear HCs through gene therapy attenuates HC injury and partially protects against neomycin‐induced HL.

To investigate the mechanism by which *Xaf1* knockdown mitigates neomycin‐induced ototoxicity, we conducted RNA‐seq on the cochlear tissues of the EGFP+Neo and Cre + Neo groups. The PCA of RNA‐seq data demonstrated pronounced transcriptional segregation between EGFP+Neo and Cre+Neo groups along the primary variance axis (Figure S6B), indicative of neomycin‐induced global transcriptomic reconfiguration. The EGFP+Neo group showed downregulation of 780 genes and upregulation of 478 genes compared to the Cre+Neo group (Figure 7I). Notably, the expression level of *Xaf1* in the Cre+Neo group dramatically declined compared to the EGFP+Neo group (Figure 7I). The key genes involved in NF‐κB signalling pathway activation and inflammatory response, such as *CD14* and *Btk*, were downregulated in the Cre+ Neo group (Figure 7J). More significantly, PANoptosis‐related genes, including *Xaf1*, *Zbp1*, *Gsdmd*, *Ripk1*, *Ripk3* and *Mlkl*, were significantly downregulated in the Cre + Neo group (Figure 7J). These results suggest that *Xaf1* knockdown reduces the expression of PANoptosis‐related genes, which ultimately weakens the inflammatory response and the damage to HCs.

Next, we investigated the mechanism by which Xaf1 drives PANoptosis. Building on prior evidence that ZBP1 and AIM2 are core components of the PANoptosome [40], we performed co‐immunoprecipitation (Co‐IP) assays in HEI‐OC1 cells of the control (Ctrl) and Xaf1 overexpression (Xaf1‐OE) groups, focusing on ZBP1 and AIM2. Notably, Co‐IP results revealed significant enrichment of ZBP1, but not AIM2, in the Xaf1‐OE group IP lysates compared to the Ctrl group (Figure S7A–B), suggesting a specific physical interaction between XAF1 and ZBP1. To further validate this regulatory relationship in vivo, we analysed ZBP1 expression in the cochleae of *Xaf1* knockdown mice following neomycin injury. Western blot results demonstrated that neomycin treatment upregulated ZBP1 protein levels in the EGFP+Neo group, whereas this induction was abolished in the Cre + Neo (*Xaf1* knockdown) group (Figure S7C). These findings align with our RNA‐seq data showing concordant dynamics of *Zbp1* transcript levels (Figures 6C and 7J). Together, these results demonstrate that Xaf1 acts upstream of ZBP1 by directly binding to ZBP1 and regulating its expression, thereby driving PANoptosis activation.

## 3 Discussion

AGs remain clinically indispensable for treating life‐threatening infections, yet their therapeutic application presents a paradoxical challenge due to severe dose‐limiting toxicities [11]. Notably, neomycin and related AGs demonstrate irreversible neurotoxicity and permanent SNHL that profoundly impact patient quality of life [8, 9]. Therefore, current research priorities have focused on developing targeted therapeutic strategies to prevent or reverse drug‐induced ototoxicity while preserving antimicrobial efficacy.

Research on the peripheral auditory system poses considerable challenges due to its cellular heterogeneity, precise cytoarchitecture and limited cell quantity [41, 42]. Conventional bulk RNA‐seq approaches are constrained by their inability to resolve cell‐type‐specific expression profiles, resulting in averaged transcriptional signatures that obscure critical biological information. This limitation is particularly problematic for studying complex sensory organs like the inner ear, where cellular specialisation and spatial organisation are paramount for auditory function. ScRNA‐seq has emerged as a revolutionary tool for high‐resolution transcriptome analysis at the single‐cell level, and this approach has revolutionised our understanding of cellular components, developmental trajectories and disease mechanisms in complex tissues and organs [41, 42]. Recent advances in scRNA‐seq have facilitated the unprecedented characterisation of cochlear cell types and their developmental paths, such as HC specification and sensory neuron bifurcation, providing critical insights into auditory organ development [24, 43].

To elucidate the molecular mechanisms underlying neomycin‐induced ototoxicity and to identify key regulatory targets in cochlear injury, we performed snRNA‐seq on cochlear tissues exposed to acute and chronic neomycin treatment. Our findings revealed cochlear cellular vulnerability patterns in which Reissner's membrane cells, HCs and Hensen's cells emerged as primary cell types involved in neomycin‐induced acute injury, while SGNs, OHCs and SV cells showed progressive vulnerability in neomycin‐induced chronic injury. Furthermore, we identified a conserved response signature across the cochlea, including the SV, HCs and SGNs, thus providing a comprehensive single‐cell transcriptomic atlas for targeted therapeutic development against aminoglycosideinduced HL.

Previous studies have established apoptosis as the predominant mechanism driving drug‐induced ototoxicity [44]. In support of this, we found that neomycin‐induced cochlear damage was associated with increased localisation of Cleaved Caspase3‐positive cells among HCs, SGNs and SV cells, indicating the activation of apoptotic pathways in these cell populations. However, we also observed that neomycin exposure triggered pyroptosis and necroptosis in HCs and SV cells, collectively resulting in PANoptosis within these cell types. Intriguingly, SGNs exclusively experienced pyroptosis and apoptosis, while necroptosis was absent. These findings suggested that the vulnerability of cochlear cells to neomycin‐induced injury exhibits cellular heterogeneity, and the occurrence of PANoptosis in HCs and SV cells has emerged as a potential major biochemical mechanism for neomycin‐mediated ototoxicity.

Prior scRNA‐seq data showed pronounced upregulation of *ZBP1*, *XAF1*, *IFI44L* and *SOCS1* in B cells from sepsis patients that displayed heightened PANoptosis sensitivity [36]. Meanwhile, our snRNA‐seq data on neomycin‐induced chronic cochlear injury showed that *Xaf1* expression was highly enriched in OHCs upon neomycin treatment. Together, these findings prompted us to investigate the potential role of Xaf1 in regulating PANoptosis. While prior studies established the pro‐apoptotic activity of Xaf1 [45, 46], its functional involvement in PANoptosis remained undefined. To address this, we overexpressed Xaf1 in HEI‐OC1 cells to analyse its contributions to the occurrence of PANoptosis. As previously reported [13], neomycin treatment significantly promoted apoptosis and increased ROS levels in HEI‐OC1 cells. Notably, the overexpression of Xaf1 similarly triggered cell pyroptosis and necroptosis, thereby replicating the hallmarks of PANoptosis. In addition, the ablation of Xaf1 in HCs in an in vitro neomycin‐induced cochlear explant injury model could markedly suppress the occurrence of PANoptosis in HCs and increase their viability. These results established Xaf1 as a pivotal regulator of neomycin‐driven PANoptosis in cochlear HCs, thus revealing its role in coordinating distinct cell death modalities. Crucially, *Xaf1* knockdown in cochlear HCs in vivo can significantly inhibit neomycin‐induced HL in mice. Our findings collectively indicated that Xaf1 plays a key role in PANoptosis in HCs during neomycin‐induced ototoxicity.

The bulk RNA‐seq analysis indicated that the upregulated genes in the neomycin‐injured cochlea are primarily related to the occurrence of PANoptosis and NF‐κB‐mediated inflammatory response genes, while *Xaf1* knockdown markedly decreased the expression of these genes. PANoptosis is an integrated cell death scaffold that converges the pyroptotic, apoptotic and necroptotic machinery through ZBP1‐mediated supramolecular complex assembly, thereby coordinating immunogenic demise via caspase activation and membrane rupture cascades [26]. Hence, PANoptosis is crucial for inflammatory cell death by promoting the production of proinflammatory cytokines and damage‐associated molecular patterns [47]. The proinflammatory cytokines and damage‐associated molecular patterns stimulate the NF‐κB signalling pathway, resulting in inflammatory substances that exacerbate cellular or tissue damage [48]. Prior research has indicated that stimuli or conditions such as drugs (gentamicin) [49], noise [50] and senescence [51] might stimulate the NF‐κB signalling pathway, thus inciting inflammatory responses and ultimately culminating in cochlear injury. Therefore, *Xaf1* knockdown primarily inhibits PANoptosis, resulting in reduced secretion of proinflammatory cytokines, which suppresses the activation of the NF‐κB signalling pathway and mitigates the inflammatory response, ultimately decreasing damage to HCs and enhancing survival. Mechanistically, Xaf1 drives PANoptosis activation by directly binding to ZBP1 and regulating its expression, positioning Xaf1 upstream of ZBP1.

In conclusion, through AAV‐mediated *Xaf1* knockdown, we demonstrated that Xaf1 inhibition significantly attenuates neomycin‐induced PANoptosis activation, HC loss and HL, revealing the critical role of Xaf1 in the pathological neomycin‐induced ototoxicity cascade. Gene therapy strategies based on conditional knockout of *Xaf1* in cochlear HCs and SV cells to inhibit the occurrence of PANoptosis is a promising treatment method for preventing AG‐induced ototoxicity.

## 4 Materials and Methods

### 4.1 Animals

*Xaf1* ^flox/flox^ mice were a gift from Prof. Jin Jin from Zhejiang University. C57BL/6J and ICR mice were purchased from Jiangsu Qinglongshan Biotechnology Co. LTD. All of our animal experiments were conducted according to protocols permitted by the Southeast University Animal Care and Use Committee (No. 20220901020), which are aligned with the National Institutes of Health Guidelines for the Care and Use of Laboratory Animals. We implemented comprehensive measures to minimise animal distress and reduce the number of animals in the study under ethical research standards.

### 4.2 10× Chromium RNA Sequencing

Cochlear explants from P3 mice in the Ctrl group (*n* = 22) and the Neo group (*n* = 22) were collected as one biological replicate and subsequently pooled for snRNA‐seq. The purified nuclei were resuspended to a final concentration of 700–1200 nuclei/μL and placed onto the Chromium Next GEM Chip. Nuclear barcoding, cDNA amplification and library preparation were performed following the 10**×** Genomics methods (CG000204_Rev D). cDNA library sequencing was then performed using an Illumina NovaSeq 6000 System at OE Biotech Co. Ltd. We used the Mm10_3.0.0 reference genome for read alignment. Gene expression matrices were produced and measured using the default parameters of the 10**×** Genomics Cell Ranger v3 pipeline (version 5.0.0). Filtered count matrices from the Cell Ranger pipeline were used for subsequent downstream analyses.

### 4.3 Quality Control of snRNA‐Seq Data and Cell‐Type Annotation

To more comprehensively investigate the pathological processes and molecular mechanisms underlying neomycin‐induced cochlear injury, we integrated our in‐house snRNA‐seq data (the acute neomycin‐induced cochlear injury model) from a chronic neomycin‐induced cochlear injury model previously reported by our group into a unified analytical framework [23]. The Scanpy Python package (v.1.9.8) was used for quality control and cell type annotation. Four gene expression matrices (P3_Ctrl, P3_Neo, P20_Ctrl and P20_Neo) were analysed. Briefly, cells expressing more than 5000 genes, fewer than 500 counts or more than 25% mitochondrial content were excluded. This stringent quality control step was essential to ensure the accuracy and reliability of the data. Following quality control, a total of 53,311 nuclei were included for reduction and clustering, comprising the following conditions: P3_Ctrl (16,366), P3_Neo (10,680), P20_Ctrl (6598) and P20_Neo (19,667). The data were subsequently adjusted using the normalize_total function and were log‐transformed using the log1p function. Highly variable genes were identified using the high_variable_genes function. We used the Leiden algorithm for unsupervised clustering and used uniform manifold approximation and projection (UMAP) for the visual representation of the dimensionality reduction. To annotate cell types within the mouse cochlea, we referenced a list of canonical marker genes previously validated by Kolla L et al. and Jean P et al. [17, 24].

### 4.4 Batch Effect Correction and Integration Analysis

SnRNA‐seq data were processed using the Scanpy toolkit (v1.10.3) following established preprocessing protocols, including quality control filtering and normalisation of the cell‐gene count matrix originally generated through Seurat conversion, along with integration of specimen metadata [52]. Integration was performed using scVI‐tools (single‐cell Variational Inference [53], v1.1.2) to align the datasets. To optimise the balance between biological variance preservation and batch effect removal, we evaluated multiple integration methods using the single‐cell integration benchmarking (scIB) approach [54]. The methods assessed included batch‐balanced k‐nearest neighbours (*BBKNN*), *Scanorama*, *Harmony*, the bulk data integration tool *ComBat*, *scVI* and *scANVI*. Each method was implemented according to the authors' default settings (additional details are provided in the shared code on GitHub). The integration quality—specifically, the degree of batch correction and biological information retention—was quantitatively assessed using scIB metrics. Based on these evaluations, *scANVI* was selected as the final method for subsequent analyses.

### 4.5 Sensitivity Analysis of Cochlear Cell Types Upon Neomycin Injury

We employed the *Augur* and *scDist* packages for perturbation analysis to assess the sensitivity of various cochlear cell types to neomycin‐induced injury. *Augur* is a technique formulated to rank cell types that exhibit the greatest responsiveness to biological perturbations in single‐cell data. In a high‐dimensional space, it measures the separability of perturbed and unperturbed cells using a machine‐learning framework [55]. The *scDist* software uses linear mixed‐effects models to account for individual and technical heterogeneity in the snRNA‐seq data while also introducing an interpretable metric for comparing different cell types [56]. In brief, we integrated *scANVI* information, batch information and neomycin exposure data to create multiple datasets in R (v.4.1.3). Using the calculate_auc function in *Augur*, we computed the performance scores for each cell group after neomycin exposure, and this was followed by visualisation with the plot_lollipop function. The *scDist* package was then used to validate the results obtained from *Augur*. Specifically, *scDist* allowed us to reanalyse the data after incorporating batch information, with data visualisation being achieved through the Distplot function.

### 4.6 Scoring of PANoptosis Pathway Activity for Cell Subpopulations

We used the *rank_genes_groups* function in Scanpy to identify DEGs between the Neo and Ctrl groups under acute or chronic conditions. Gene sets with significant expression changes under both conditions were selected based on an adjusted *p*‐value threshold (p_adj < 0.001). GO and KEGG gene sets were used in Gene Set Enrichment Analysis (GSEA) to identify PANoptosis genes, with further screening based on relevant research [57]. Next, we measured the genes in the Ctrl and Neo groups using the gene scoring approach. This analysis measured pathway activity in response to neomycin treatment compared to the Ctrl group.

### 4.7 Bulk RNA Sequencing and Data Analysis

*Xaf1* ^flox/flox^ mice (P1) were subjected to round window injections of AAV‐EGFP or AAV‐Cre. Subcutaneous administration of neomycin (125 mg/kg) commenced on P7 and persisted until P14, and this was followed by a control injection of physiological saline. Cochlear samples were obtained from all animals at P30 for bulk RNA sequencing. Following RNA sequencing and genome data matching, gene expression matrices were derived for all samples. We used the R package edgeR [58] to examine the RNA‐seq data to identify DEGs across multiple groups, and we chose significant DEGs based on a P‐value < 0.05 and | log2Fold Change | ≥ 0.25. The DEGs were then used for GO and KEGG analysis via the *Metascape* platform [59].

### 4.8 Cell Experiments

The 3× Flag‐ *Xaf1* plasmid was constructed by Shanghai Taitool Bioscience Co. Ltd., and the HEI‐OC1 cell line was used for investigating both the protective and damaging effects on HCs. HEI‐OC1 cells were cultured at 37°C in a 5% CO_2_ incubator in DMEM (Gibco, 11,995,500) supplemented with 10% fetal bovine serum (FBS, Vazyme, F101) and 1% ampicillin (Beyotime, ST008). Transfection of the 3× FLAG‐ *Xaf1* plasmid into HEI‐OC1 cells was performed using Opti‐MEM (Gibco, 31,985,062) and Lipofectamine 2000 (Invitrogen, 11,668,027) when the cell confluence reached 70%–80%. After a 6 h incubation, the transfection medium was substituted with regular culture medium, and the cells were grown for an additional 48 h. At this time, cells were harvested for subsequent analyses, including flow cytometry, Western blotting, Co‐IP, RT‐qPCR and immunofluorescence.

### 4.9 Cochlear Explant Experiment

Cochleae were dissected from P3 wild‐type C57BL/6J mice in cold phosphate‐buffered saline (PBS) and adhered to cell slides (Biosharp, BS‐09‐RC) coated with Cell‐Tak (Corning, 354,240). Cochlear explants were cultured in advanced DMEM/F‐12 medium (Gibco, 12,634,010) enriched with N2 (1%, Gibco, 17,502,048), B‐27 (2%, Gibco, 17,504,044), IGF (50 ng/mL, Sigma, I8779), FGF (10 ng/mL, Sigma, F0291), EGF (20 ng/mL, Sigma, E9644) and ampicillin (1%, Beyotime, ST008) at 37°C and 5% CO_2_. After a 6 h recovery period, the cochleae were treated with 0.5 mM neomycin for 12 h to induce cochlear damage.

### 4.10 Genotyping

Genomic DNA was isolated from the tail tips of *Xaf1* ^flox/flox^ mice by lysing tissue samples in 50 mM NaOH at 98°C for 30 min, followed by neutralisation with 1 M Tris–HCl (pH 8.0, Solarbio, T1150). The following components were included in the genotyping PCR reaction mixture: 5 μL PCR mix (Vazyme, p131‐02), 1.5 μL genomic DNA, 1 μL primers and 2.5 μL water. Following a three‐minute denaturation step at 94°C, the amplification technique consisted of 38 cycles of 30 s each at 94°C, 58°C and 72°C for annealing and extension, respectively. *Xaf1*‐specific primers were used for the PCR. The primer sequence for *Xaf1* was: *AAG CCT AAC GCA TCT CCT CGG* (Forward primer), *TTC ATT TGG GCA AAG GAC TA* (Reverse primer).

### 4.11 AAV Injection

To knock down *Xaf1* in cochlear HCs, Cre AAV was administered by injection into the cochlear round window membrane of *Xaf1* ^flox/flox^ mice. Briefly, *Xaf1* ^flox/flox^ mice (P1) were anaesthetised by immersion in ice for 2 min. A volume of 1.5 μL of AAV virus (AAV‐PHP.eB[ssAAV.CAG.EGFP‐2A‐Cre.WPRE.SV40pA] or AAV‐PHP.eB[ssAAV.CAG.EGFP‐2A. WPRE.SV40pA], acquired from PackGene Biotech) was administered through the round window membrane of the left ear using a glass micropipette (25 μm) regulated by a microelectrode puller (MP500, RWD). The incision was sealed using tissue adhesive (3 M Vetbond, 1469SB). After the surgery, the mice were placed on a 37°C thermostatic heating pad for 10 min before being returned to their mother for further nursing.

### 4.12 Neomycin‐Induced Chronic Ototoxicity Model

A chronic ototoxicity model was established in C57BL/6J mice through subcutaneous administration of neomycin. Mice received daily subcutaneous injections of neomycin (150 mg/kg or 125 mg/kg) for eight consecutive days (P7 to P14). Neomycin was solubilised in sterile saline to a final concentration of 15 mg/mL, and a dosage of 0.1 mL per 10 g of body weight was delivered. Hearing thresholds were assessed at P30 by ABR tests.

### 4.13 ABR Tests

ABR tests were conducted using TDT audiometry equipment (RZ6, Tucker‐Davis Technologies) and *BioSigRZ* software. P30 mice were anaesthetised with 1.25% tribromoethanol (0.2 mL/10g, intraperitoneal injection), and then recording electrodes were positioned along the midline of the skull, with reference and ground electrodes implanted behind each ear. Tone bursts at frequencies of 4, 8, 12, 16, 24 and 32 kHz were used as stimuli. The stimulation level started at 90 dB SPL and was decreased in 5 dB increments until no repeatable ABR waveforms were observed. Mice were kept on heating pads until fully recovered and then returned to the breeding facility. Both open‐field and closed‐field ABR testing were performed using the same setup.

### 4.14 Flow Cytometry

Annexin V‐FITC (C1062S, Beyotime) is frequently used for detecting apoptosis, while propidium iodide (PI) is used to differentiate between viable and non‐viable cells. HEI‐OC1 cells were subjected to digestion with 0.25% EDTA‐free trypsin for 3 min, followed by centrifugation at 1000 × *g* for 5 min to harvest the cells. The cell aggregates were rinsed three times with 1× PBS. After that, 1 × 10^5^ cells were resuspended in 195 μL of Annexin V binding buffer, and 10 μL of PI solution and 5 μL of Annexin V‐FITC were added. The cells were gently mixed and then incubated for 20 min at room temperature in the dark. The ratios of FITC‐positive and PI‐positive cells were evaluated by flow cytometry (Cytoflex, Beckman, CA, USA) within one hour.

The generation of mitochondrial ROS was measured using 2′,7‐Dichlorodihydrofluorescein diacetate (DCFH‐CA) (D6883, Sigma‐Aldrich). After the removal of the HEI‐OC1 cell culture media, the cells were rinsed two times with cold 1× PBS. The adhering cells were subsequently treated with DCFH‐CA solution in a 37°C incubator in the dark for 20 min. After incubation, the cells were washed three times with 1× PBS, treated with warmed 0.25% EDTA‐free trypsin for digestion, centrifuged and resuspended in 1× PBS. ROS levels were measured by flow cytometry (Cytoflex, Beckman, CA, USA) based on green fluorescence.

### 4.15 RT‐qPCR

Total RNA was extracted from mouse cochleae and HEI‐OC1 cells using Trizol Reagent (15596–018, Invitrogen). The RNA concentration was measured using a Nanodrop spectrophotometer (Thermo Fisher), and the RNA was then reverse transcribed into cDNA using a reverse transcription kit (R333, Vazyme). RT‐qPCR was conducted using the 2× Taq Pro‐Universal SYBR qPCR Master Mix (Q712, Vazyme) on an Applied Biosystems CFX96 real‐time PCR system (Bio‐Rad). The internal control for mRNA expression normalisation was GAPDH. We used the 2^−∆∆CT^ method to calculate the levels of gene expression. The sequences of the RT‐qPCR primers are provided in Table S1.

### 4.16 Western Blot

For total protein extraction, mouse cochleae and HEI‐OC1 cells were lysed in RIPA lysis buffer (P0013C, Beyotime) enhanced with a protease inhibitor cocktail (04693132001, Roche). Protein samples were centrifuged, combined with 5× SDS loading buffer (LT101, Epizyme), and heated for 10 min at 98°C. Following SDS‐PAGE separation of the protein samples using a PAGE Gel Rapid Preparation Kit (12.5%, PG113, Epizyme), the samples were transferred to a PVDF membrane (ISEQ00010, Millipore) and blocked for one hour at room temperature using 5% (v/v) skim milk in 1× TBST (1× Tris‐buffered saline containing 0.1% Tween‐20). Primary antibodies were incubated with membranes overnight at 4°C. The antibodies used are detailed in Table S2. The membranes were subsequently incubated with HRP‐conjugated secondary antibodies (M21001, M21002, Abmart) for one hour at ambient temperature, followed by three 10 min washes with 1× TBST. Protein bands were visualised using a Tanon‐5200 imaging system (Tanon, China).

### 4.17 Immunofluorescence

Immunostaining was performed on both cultured cochlear explants and adult mouse tissues. Before blocking, samples of cultured cochleae were fixed in 4% paraformaldehyde (PFA) for one hour at room temperature. For cross‐sectional samples, the cochleae were fixed in 4% PFA for 24 h, decalcified in 0.5 M EDTA for an additional 24 h at 4°C and dissected into the apical, middle and basal turns in cold PBS. For vertical sections, cochleae were fixed, decalcified and then gradually dehydrated in sucrose solution, embedded in OCT and sectioned at 14 μm thickness. The cochlear sections were blocked for 2 h at room temperature, incubated overnight with primary antibodies and incubated for 1 h at room temperature with secondary antibodies. Finally, the samples were mounted in DAKO mounting medium (Dako, S3023). The HEI‐OC1 cells were stained for immunofluorescence following the same steps. The antibodies used are detailed in Table S2.

### 4.18 Co‐Immunoprecipitation

The HEI‐OC1 cells of the Ctrl and Xaf1‐OE groups were lysed in RIPA buffer supplemented with protease and phosphatase inhibitors. Lysates were centrifuged at 13,000 × g for 30 min and incubated overnight at 4°C with Anti‐Flag Magnetic Beads (Beyotime, P2115). Beads were washed three times with lysis buffer, and bound proteins were eluted in 1× SDS loading buffer, followed by boiling at 100°C for 10 min. Eluates were resolved by SDS‐PAGE and immunoblotted with anti‐ZBP1 (1:100, Cell Signalling Technology, 33,402), anti‐AIM2 (1:100, Proteintech, 20,590‐1‐AP), anti‐Flag (1:1000, Abmart, M20008) and anti‐GAPDH(1:5000, Proteintech, 10,494‐1‐AP) antibodies.

### 4.19 Statistical Analysis

Statistical analysis for snRNA‐seq and bulk RNA‐seq was described in the detailed methods. This work used GraphPad Prism 8 software for data processing and visualisation, with all statistical data presented as the mean ± standard error of the mean. Unpaired Student's t‐test was used to compare the differences between two datasets, and one‐way ANOVA was used to examine the differences between multiple datasets.

## Author Contributions

**X.W.:** research design, collation and analysis of the experimental results, manuscript writing. **H.X.:** AAV injection, validation experiment of cochlear explants, mouse‐related validation experiments. **J.W.:** SnRNA‐seq data integration, analysis and visualisation. **Y.L.:** HEI‐OC1 cell line‐related validation experiments. **Y.A.:** mouse‐related validation experiments. **Z.Y.** and **X.T.:** basic experiments. **F.K.:** bulk RNA‐seq data analysis and visualisation. **X.C.:** writing review and editing, project administration, funding acquisition. **R.C.:** resources, project administration, funding acquisition. **S.Z.:** supervision, project administration, funding acquisition, writing review and editing. All authors read and approved the final manuscript.

## Conflicts of Interest

The authors declare no conflicts of interest.

## Supporting information


**Data S1.** Supporting Information.

## Data Availability

The data underpinning the findings of this investigation can be obtained from the authors upon reasonable request. All custom notebooks and scripts used in this study have been deposited at https://github.com/Shinnyee/code_Xaf1.
