# Supplementary material for: Single‐Nucleus Transcriptomics Uncovers Xaf1‐Driven PANoptosis as a Therapeutic Target in Aminoglycoside‐Induced Hearing Loss
Source: Cell Prolif. 2025 Jul 2;59(1):e70081. doi: 10.1111/cpr.70081 (PMC12774624; doi:10.1111/cpr.70081)
Supplement: Supplementary file 1 — Data S1. Supporting Information. [file CPR-59-e70081-s001.pdf]

Supplementary Materials

Single-nucleus transcriptomics uncovers Xaf1-driven PANoptosis as a therapeutic target in aminoglycoside-induced hearing loss

Xinlin Wang[1 †], Hairong Xiao[1, 6 †], Jiheng Wu[1 †], Yanqin Lin[1, 6 †], Yiheng Ao[1 †], Zixuan Ye[1], Xin Tan[1], Fanliang Kong[1], Xin Chen[1, 7, #], Renjie Chai[1, 2, 3, 4, 5, 6, #], Shasha Zhang[1, 6, #]

List contents for supplementary materials

1. Supplementary Figures 1-7
2. Supplementary Tables 1-2

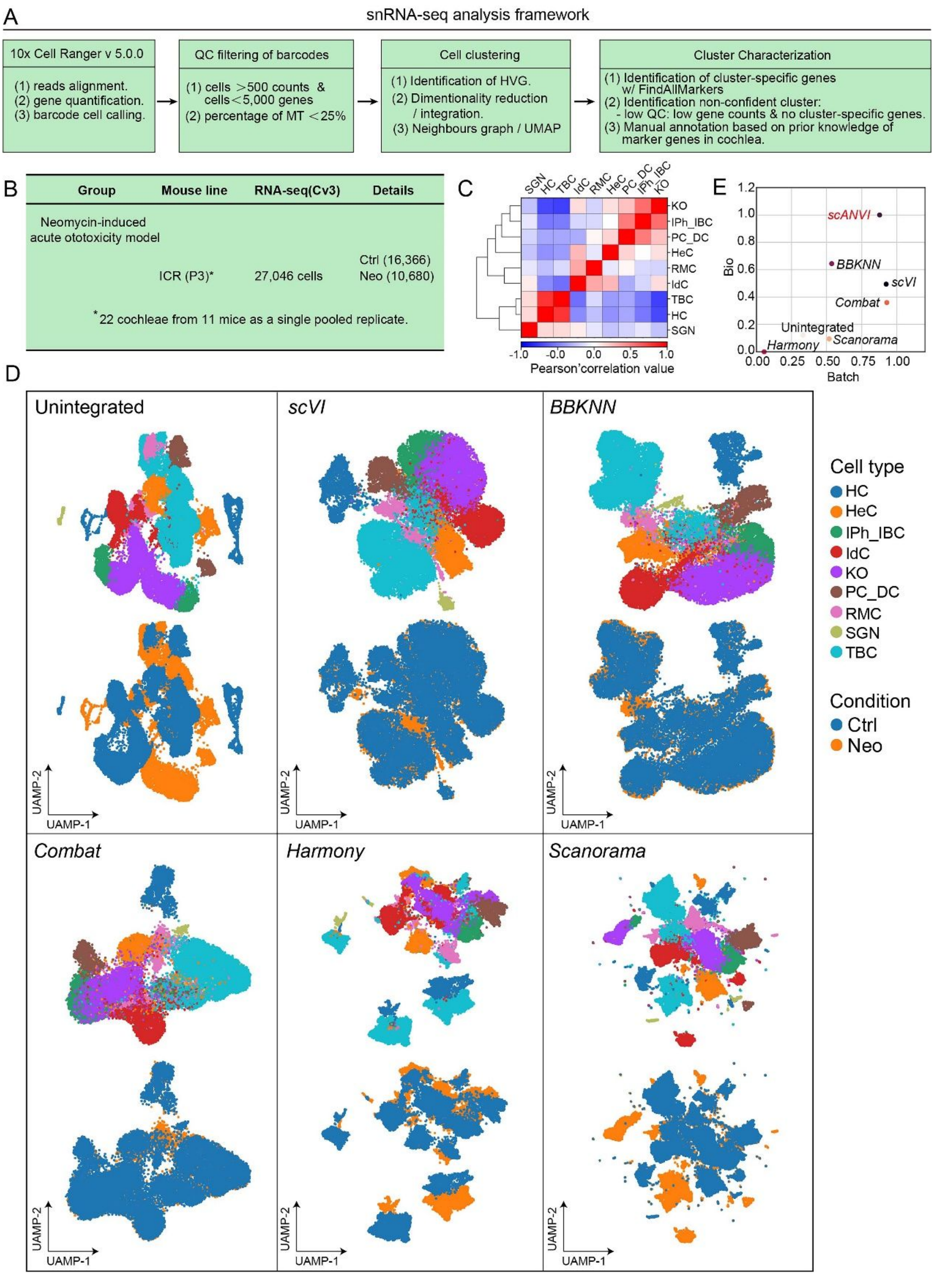

Figure S1. Single-nucleus transcriptomic profiling revealed cochlear cell type-specific vulnerability to the neomycin-induced acute ototoxicity model. (A) The analysis framework of snRNA-seq data quality control and analysis is shown in a flowchart. **(B)** The details of the snRNA-seq samples from mice with a neomycin-induced acute injury model. **(C)** The heatmap displayed the correlation (with Pearson's

correlation value) between different cell groups in the sensory epithelium of neomycin-induced acute injury. **(D)** The UAMP showed that *scVI*, *BBKNN*, *ComBat*, *Harmony*, and *Scanorama* algorithms integrate the clustering and distribution of cell types in the Ctrl and Neo groups. **(E)** The wireframe chart showed the scores of different algorithms for removing batches between samples while retaining biological significance.

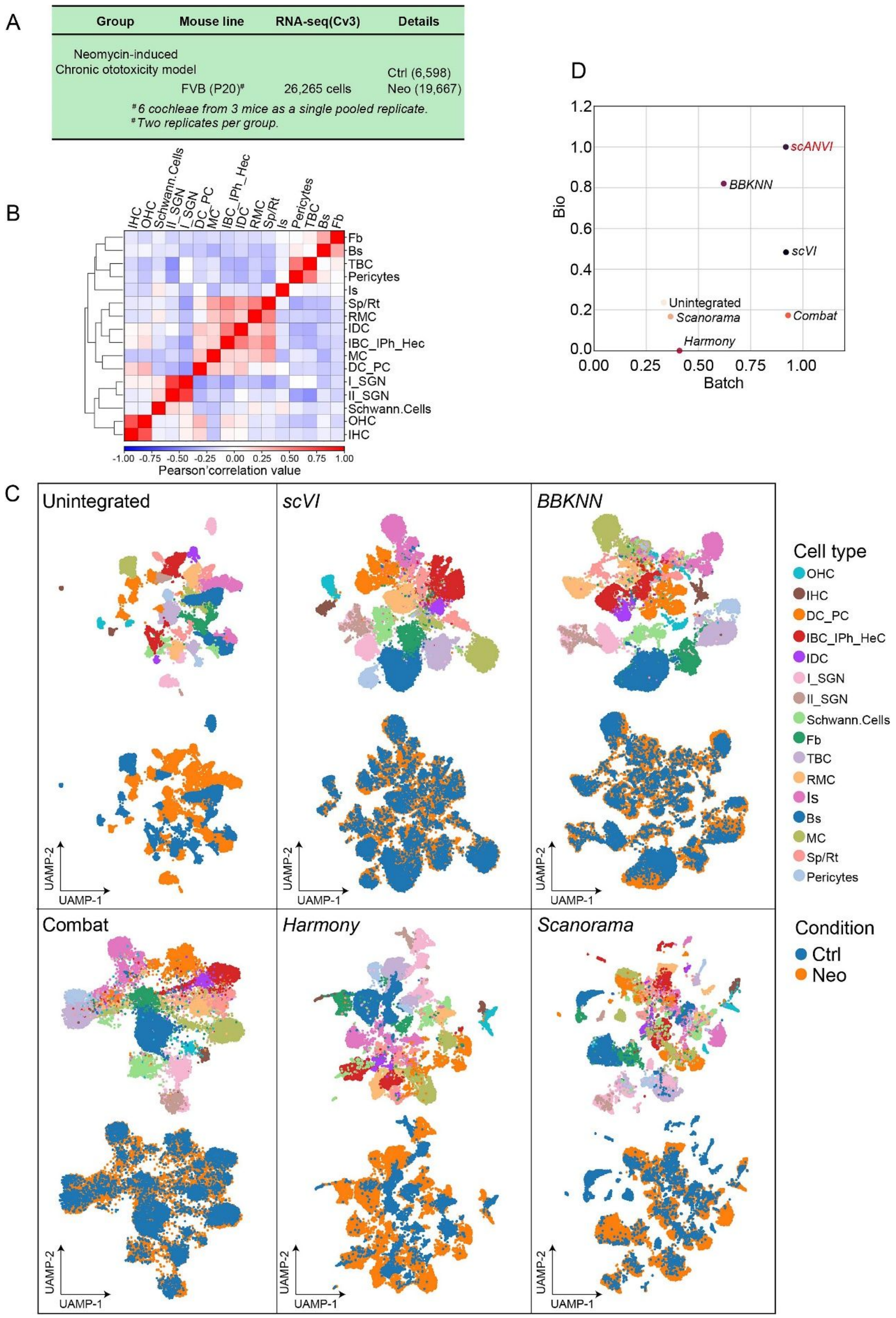

Figure S2. Single-nucleus transcriptomic profiling revealed cochlear cell type-specific vulnerability to the neomycin-induced chronic ototoxicity model. (A) The details of the snRNA-seq samples from mice with a neomycin-induced chronic injury

model. **(B)** The heatmap displayed the correlation (with Pearson's correlation value) between different cell groups in the cochlea of neomycin-induced chronic injury. **(C)** The UAMP showed that *scVI*, *BBKNN*, *ComBat*, *Harmony*, and *Scanorama* algorithms integrate the clustering and distribution of cell types in the Ctrl and Neo groups. **(D)** The wireframe chart showed the scores of different algorithms for removing batches between samples while retaining biological significance.

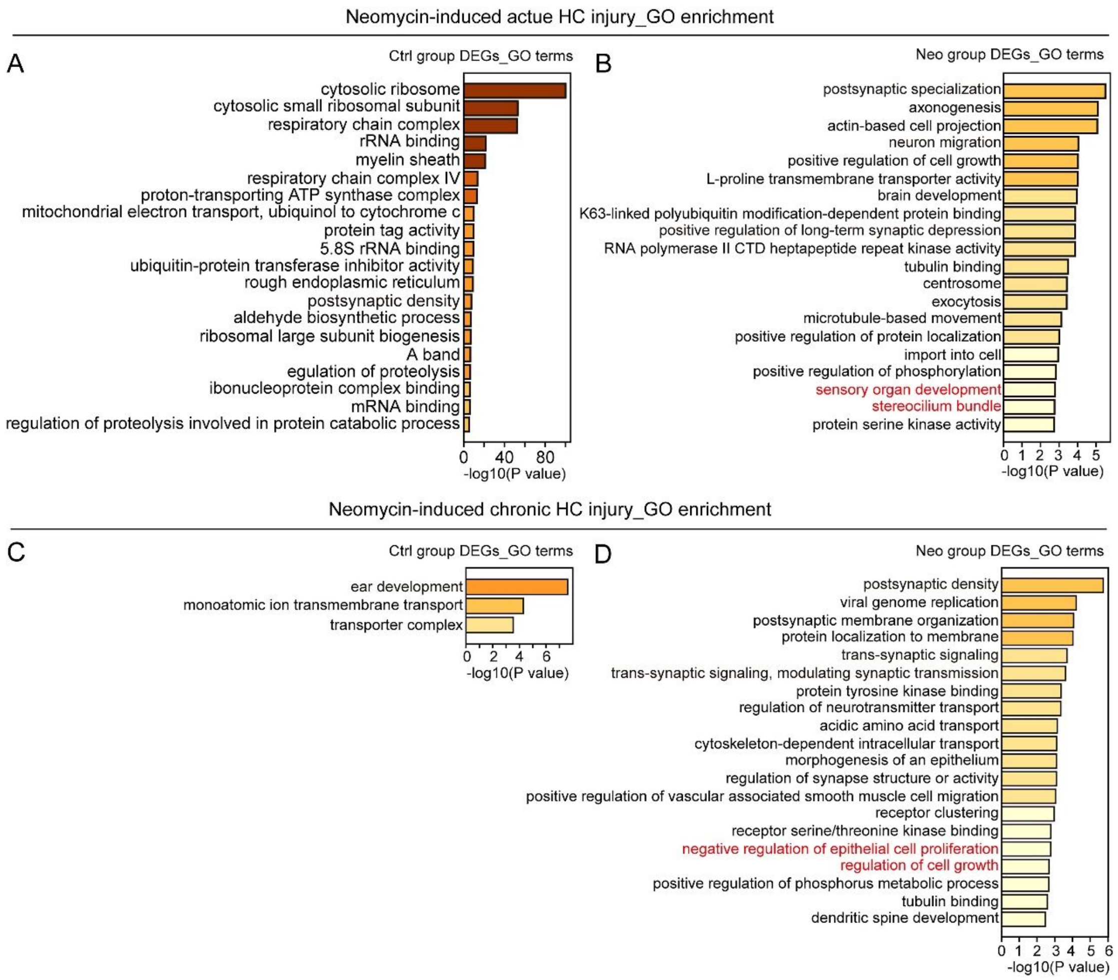

Figure S3. GO enrichment analysis of neomycin-induced HC injury. (A-B) GO enrichment of differentially expressed genes (DEGs) in HCs under acute neomycin injury, comparing biological processes enriched in the control group (Ctrl, A) versus the neomycin-treated group (Neo, B). **(C-D)** GO enrichment of DEGs in HCs under chronic neomycin injury, comparing processes enriched in Ctrl (C) versus Neo (D).

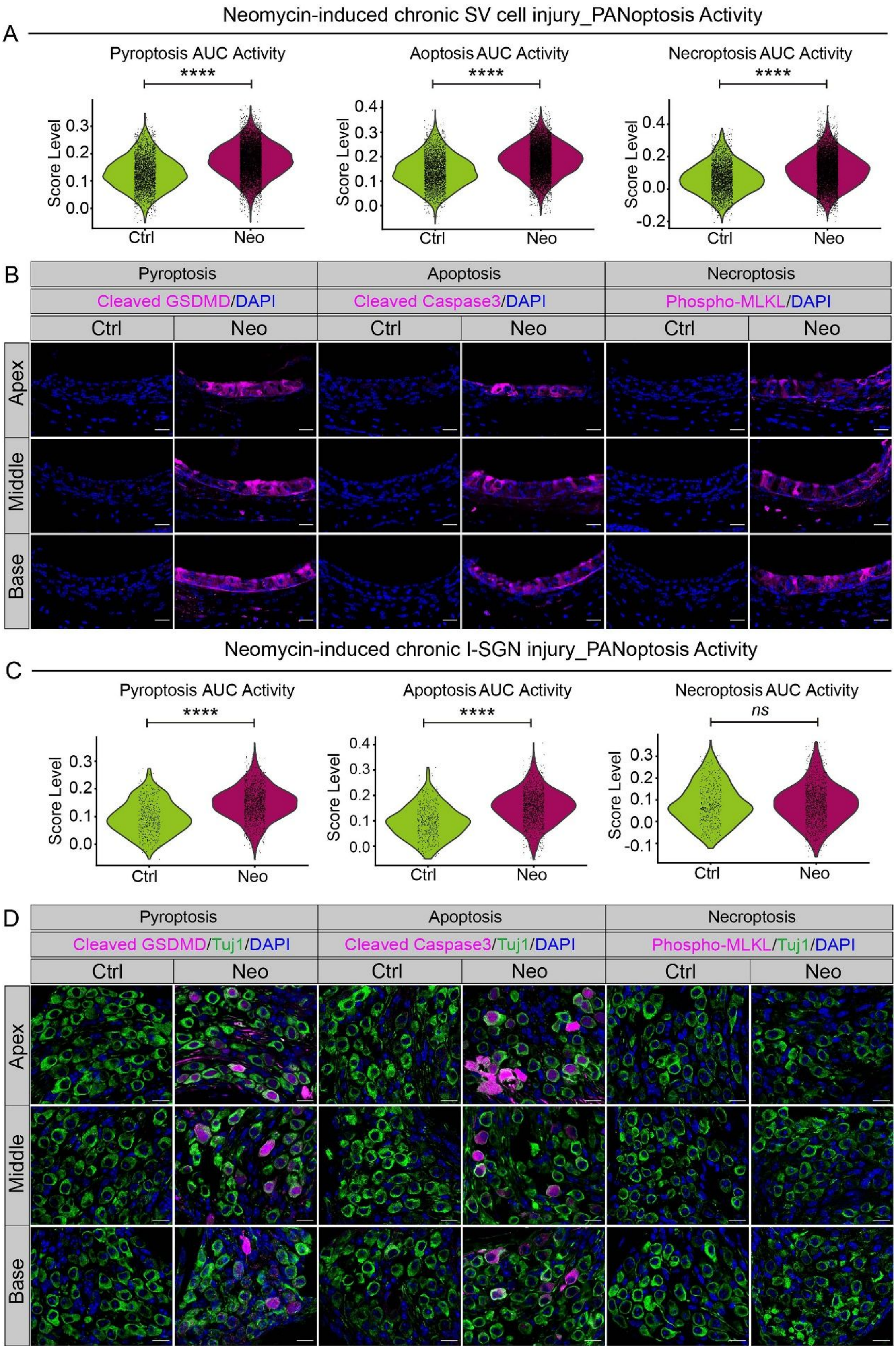

Figure S4. The cell death patterns of SGNs and SV cells in the neomycin-induced chronic ototoxicity model. (A) The violin plots depicted AUC scores for PANoptosis

pathways (pyroptosis, apoptosis, necroptosis) in the Ctrl and Neo groups from snRNA-seq analysis of neomycin-induced damaged SV cells. **(B)** Cross-sectional immunofluorescence of cochlear tissues showed SV cells expressing PANoptosis executioners in the Ctrl and Neo groups. Scale bars, 20 μm. **(C)** Same as in (A), but for neomycin-induced damaged I-SGNs. **(D)** Cross-sectional immunofluorescence of cochlear tissues showed Tuj1^{+} I-SGNs co-expression of PANoptosis with the execution of necroptosis in the Ctrl and Neo groups. Scale bars, 15 μm. *ns*, no significance. $^{****}P < 0.0001$.

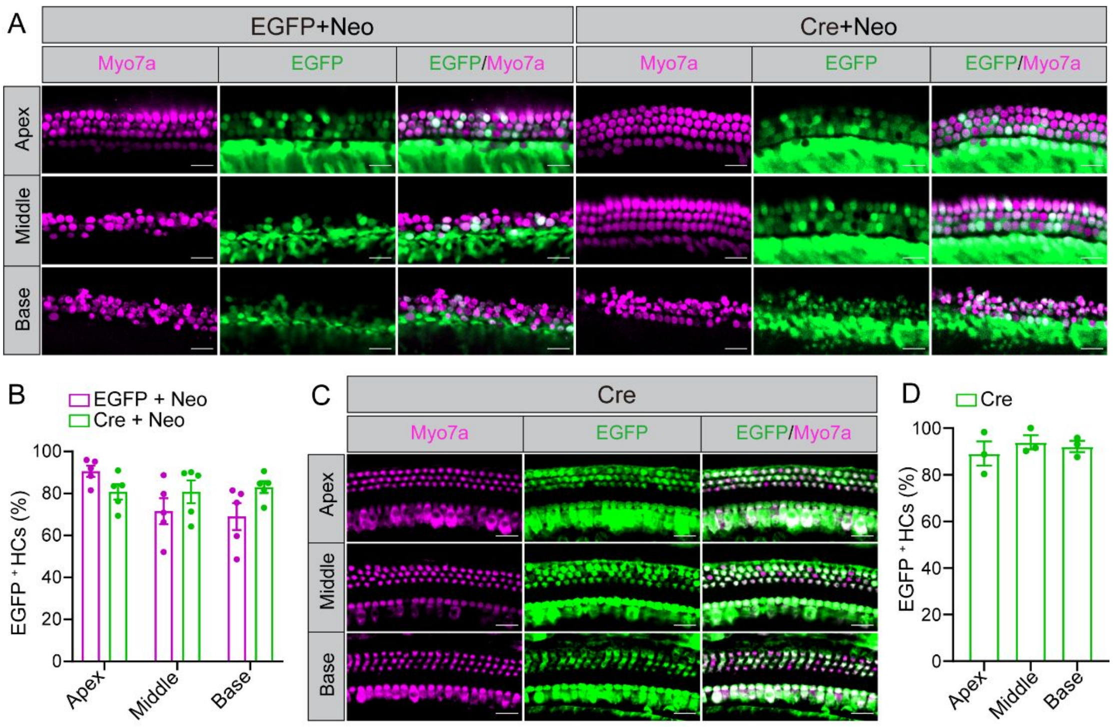

Figure S5. AAV transduction efficiency in mouse cochlea *in vitro* and *in vivo*. (A) 1.5 µL of AAV expressing either AAV-EGFP or AAV-Cre was injected into the left inner ear of *Xaf1*$^{flox/flox}$ mice (P1). The cochleae were dissected at P3 for explant culture and fixed for immunostaining. EGFP (green) was used to identify the transduced HCs. **(B)** The proportion of AAV-positive HCs per 100 µm corresponds to (A). **(C)** 1.5 µL of AAV-Cre was injected into the left inner ear of P1 *Xaf1*$^{flox/flox}$ mice (P1). Cochleae were dissected at P30 and fixed for immunostaining. EGFP (green) was used to identify the transduced HCs. **(D)** The proportion of AAV-positive HCs per 100 µm corresponds to (C). Scale bar, 20 µm. (*n* = 3).

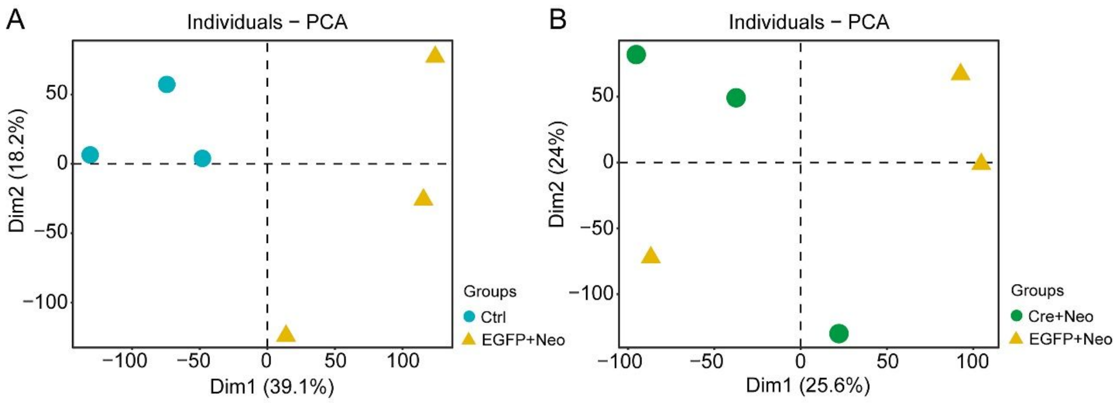

Figure S6. Principal component analysis of RNA-Seq data visualizes the intergroup differences. (A) Comparative principal component analysis (PCA) of transcriptional profiles between the Ctrl and EGFP+Neo groups. **(B)** PCA-based sample clustering analysis comparing the EGFP+Neo versus Cre+Neo experimental conditions.

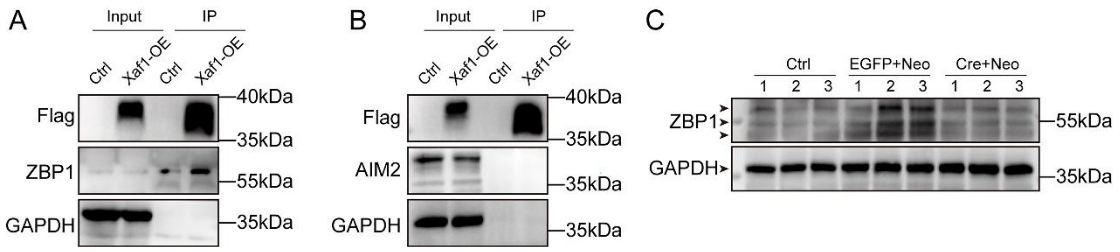

Figure S7. Xaf1 physically interacts with ZBP1 and regulates its expression to promote PANoptosis. (A-B) Co-immunoprecipitation (Co-IP) assays in HEI-OC1 cells of the Ctrl and Xaf1-OE groups demonstrated specific enrichment of ZBP1 (but not AIM2) in Xaf1-OE group IP lysates compared to Ctrl group. **(C)** Western blot analysis revealed neomycin-induced ZBP1 upregulation in the EGFP+Neo group, with abrogation of this response in the Cre+Neo (*Xaf1* knockdown) group.

Supplementary Table 1: The primer sequences for RT-qPCR.

Gene	Sequence (5'-3')
β-actin Forward	ACG GCC AGG TCA TCA CTA TTG
β-actin Reverse	AGG GGC CGG ACT CAT CGT A
Xaf1 Forward	GTG CAA AGG GCA AGC CTA AG
Xaf1 Reverse	GGC CG AAC ACT GTT TCA TGT
Bax Forward	TGA AGA CAG GGG CCT TTT TG
Bax Reverse	AAT TCG CCG GAG ACA CTC G
Caspase3 Forward	CTG ACT GGA AAG CCG AAA CTC
Caspase3 Reverse	CGA CCC GTC CTT TGA ATT TCT
Caspase7 Forward	GGA CCG AGT GCC CAC TTA TC
Caspase7 Reverse	TCG CTT TGT CGA AGT TCT TGT T
Caspase9 Forward	TCC TGG TAC ATC GAG ACC TTG
Caspase9 Reverse	AAG TCC CTT TCG CAG AAA CAG

Supplementary Table 2: The antibodies for Western blot and immunofluorescence

Antibody	Source	Cat#	Application
Anti-XAF1	Abcam	ab17204	WB/IF
DYKDDDDK-Tag (3B9) mAb	Abmart	M20008	WB
Anti-GSDMD	Abcam	ab209845	WB
Anti-GSDME	Abcam	ab215191	WB
Anti-NLRP3	Abcam	ab263899	WB
Cleaved Caspase3 (D175)	Cell Signaling Technology	9661S	WB/IF
PARP	Cell Signaling Technology	9542T	WB
Cleaved Caspase-1(Asp296) (E2G2I)	Cell Signaling Technology	89332S	WB
Cleaved Gasdermin D(Asp275) (E7H9G)	Cell Signaling Technology	36425S	IF
Phospho-MLKL (Ser345) (D6E3G)	Cell Signaling Technology	37333S	IF
Caspase 9	Cell Signaling Technology	9502s	WB
Bax	Santa Cruz	sc23959	WB
Anti-β-Actin antibody	Abcam	ab119716	WB
ZBP1	Cell Signaling Technology	33402	Co-IP/WB
AIM2 Polyclonal antibody	Proteintech	20590-1-AP	Co-IP/WB
GAPDH Polyclonal antibody	Proteintech	10494-1-AP	WB

IF: immunofluorescence; WB: Western blot; Co-IP: Co-immunoprecipitation.
